# HERQ-9 Is a New Multiplex PCR for Differentiation and Quantification of All Nine Human Herpesviruses

**DOI:** 10.1128/mSphere.00265-20

**Published:** 2020-06-24

**Authors:** Lari Pyöriä, Maija Jokinen, Mari Toppinen, Henri Salminen, Tytti Vuorinen, Veijo Hukkanen, Constanze Schmotz, Endrit Elbasani, Päivi M. Ojala, Klaus Hedman, Hannamari Välimaa, Maria F. Perdomo

**Affiliations:** a Department of Virology, University of Helsinki, Helsinki, Finland; b Department of Evolutionary Biology and Environmental Studies, University of Zürich, Zürich, Switzerland; c Institute of Biomedicine, University of Turku, Turku, Finland; d Clinical Microbiology, Turku University Hospital, Turku, Finland; e Translational Cancer Medicine Research Program, Faculty of Medicine, University of Helsinki, Helsinki, Finland; f Department of Infectious Diseases, Imperial College London, London, United Kingdom; g Helsinki University Hospital, HUSLAB, Helsinki, Finland; h Department of Oral and Maxillofacial Surgery, Helsinki University Hospital, Helsinki, Finland; University of Arizona

**Keywords:** Epstein-Barr virus, HHV-6, coinfection, cytomegalovirus, diagnostics, human herpesviruses, multiplex, qPCR, quantitative methods, tonsils, viral persistence, virome

## Abstract

Infections with the nine human herpesviruses (HHVs) are globally prevalent and characterized by lifelong persistence. Reactivations can potentially manifest as life-threatening conditions for which the demonstration of viral DNA is essential. In the present study, we developed HERQ-9, a pan-HHV quantitative PCR designed in triplex reactions to differentiate and quantify each of the HHV-DNAs: (i) herpes simplex viruses 1 and 2 and varicella-zoster virus; (ii) Epstein-Barr virus, human cytomegalovirus, and Kaposi’s sarcoma-associated herpesvirus; and (iii) HHV-6A, -6B, and -7. The method was validated with prequantified reference standards as well as with mucocutaneous swabs and cerebrospinal fluid, plasma, and tonsillar tissue samples. Our findings highlight the value of multiplexing in the diagnosis of many unsuspected, yet clinically relevant, herpesviruses. In addition, we report here frequent HHV-DNA co-occurrences in clinical samples, including some previously unknown. HERQ-9 exhibited high specificity and sensitivity (LOD_95_s of ∼10 to ∼17 copies/reaction), with a dynamic range of 10^1^ to 10^6^ copies/μl. Moreover, it performed accurately in the coamplification of both high- and low-abundance targets in the same reaction. In conclusion, we demonstrated that HERQ-9 is suitable for the diagnosis of a plethora of herpesvirus-related diseases. Besides its significance to clinical management, the method is valuable for the assessment of hitherto-unexplored synergistic effects of herpesvirus coinfections. Furthermore, its high sensitivity enables studies on the human virome, often dealing with minute quantities of persisting HHVs.

**IMPORTANCE** By adulthood, almost all humans become infected by at least one herpesvirus (HHV). The maladies inflicted by these microbes extend beyond the initial infection, as they remain inside our cells for life and can reactivate, causing severe diseases. The diagnosis of active infection by these ubiquitous pathogens includes the detection of DNA with sensitive and specific assays. We developed the first quantitative PCR assay (HERQ-9) designed to identify and quantify each of the nine human herpesviruses. The simultaneous detection of HHVs in the same sample is important since they may act together to induce life-threatening conditions. Moreover, the high sensitivity of our method is of extreme value for assessment of the effects of these viruses persisting in our body and their long-term consequences on our health.

## Abstract (precis)

By adulthood, almost all humans become infected by at least one herpesvirus (HHV). The maladies inflicted by these microbes extend beyond the initial infection, as they remain inside our cells for life and can reactivate, causing severe diseases. The diagnosis of active infection by these ubiquitous pathogens includes the detection of DNA with sensitive and specific assays. We developed the first quantitative PCR assay (HERQ-9) designed to identify and quantify each of the nine human herpesviruses. The simultaneous detection of HHVs in the same sample is important since they may act together to induce life-threatening conditions. Moreover, the high sensitivity of our method is of extreme value for assessment of the effects of these viruses persisting in our body and their long-term consequences on our health.

## INTRODUCTION

The nine human herpesviruses (HHVs) are ubiquitous pathogens that persist lifelong after primary infection. HHVs cause many disorders, ranging from mild mucocutaneous diseases to severe central nervous system conditions, birth defects, and cancer. Their ability to reactivate poses significant risks, particularly to immunosuppressed patients, such as transplant recipients, in whom Epstein-Barr virus (EBV), human cytomegalovirus (HCMV), HHV-6B, and Kaposi’s sarcoma-associated herpesvirus (KSHV) can induce life-threatening conditions (1–8).

The investigation of active HHV infections includes, among other markers, the detection of viral nucleic acids, typically by quantitative PCR (qPCR). In addition, the simultaneous detection of these pathogens has been shown to be beneficial, as their recognition may be difficult based on the clinical presentation alone (9–15).

While several multiplex qPCRs have been introduced for detection of HHVs (16–19), none are designed to quantify them all. In addition, only few of the existing protocols distinguish between the closely related HHV-6A and HHV-6B, a distinction that may be crucial, as the former still lacks clear association to disease (20–23).

In the present study, we developed a pan-herpes multiplex assay, HERQ-9, that quantifies and discriminates each of the HHVs using three separate triplex-qPCRs: the first amplifies herpes simplex viruses 1 and 2 (HSV-1 and -2) and varicella-zoster virus (VZV), the second EBV, HCMV, and KSHV, and the third HHV-6A, -6B, and -7. We validated our assay using prequantified reference materials and evaluated its performance with various clinical samples as well as solid tissue material.

HERQ-9 simplifies diagnosis and improves the clinical management and risk assessment of highly susceptible populations (1, 5, 8, 24–26). Moreover, its high sensitivity is of significant value for studies on the impact of HHV persistence on health (27, 28) and disease (29, 30).

## RESULTS

### *In silico* evaluation of amplicons, primers, and probes.

The designed primers and probes were queried against all available sequences (full or partial genomes) in the NCBI database. The oligonucleotides showed perfect match for the different strains except for four sequences of HHV-6A (GenBank accession numbers KY316054.1, KT355575.1, KY316056.1, and KY316047.1) and two of HCMV (GenBank accession numbers KY490070.1 and KP745685.1) for which one to two mismatches were observed far from the 3′ end.

We found no nonspecific binding to other viruses or human DNA except for the primers and probe of HSV-2, which also had complete homology to chimpanzee alpha-1 herpesvirus (GenBank accession number JQ360576.1).

*In silico* analysis of amplicons, primers, and probes revealed no relevant secondary structures, primer-dimers, or cross-dimers (see Fig. S1 and S2 in the supplemental material).

10.1128/mSphere.00265-20.3 FIG S1 Primer dimer, cross-dimer, and secondary-structure analysis of the primers and probes with Multiple Primer Analyzer (Thermo Fisher Scientific). The sensitivity of three was used with a primer concentration of 0.5 μM and a salt concentration of 50 mM. Download FIG S1, PDF file, 0.1 MB. Copyright © 2020 Pyöriä et al. 2020 Pyöriä et al. This content is distributed under the terms of the Creative Commons Attribution 4.0 International license.

10.1128/mSphere.00265-20.4 FIG S2 Amplicon secondary structure analyzed with the Mfold web server. The reaction temperature was set to 60°C, [Na^+^] to 50 mM, and [Mg^2+^] to 3.0 mM, and other settings were left as default. The green nucleotides represent probe binding areas. Download FIG S2, PDF file, 2.5 MB. Copyright © 2020 Pyöriä et al. 2020 Pyöriä et al. This content is distributed under the terms of the Creative Commons Attribution 4.0 International license.

### Analytical sensitivities and specificities.

We evaluated the sensitivities using eight replicates of the respective plasmids in seven dilutions ranging from 50 copies to 1 copy per reaction. Based on probit link function, the limits of detection (LOD_95_) of HERQ-9 for HSV-1, HSV-2, VZV, EBV, HCMV, KSHV, HHV-6A, HHV-6B, and HHV-7 were, respectively, 12, 13, 13, 10, 16, 17, 11, 11, and 11 copies per reaction. The values were similar in the singleplex format (see Fig. S3 in the supplemental material).

10.1128/mSphere.00265-20.5 FIG S3 Probit link function estimating LOD_95_ of qPCRs in singleplex and multiplex formats. The proportion of positives from eight plasmid replicates at 50, 25, 15, 10, 5, 3, and 1 copy/reaction was fit into the model to approximate the assay sensitivity. Download FIG S3, PDF file, 0.2 MB. Copyright © 2020 Pyöriä et al. 2020 Pyöriä et al. This content is distributed under the terms of the Creative Commons Attribution 4.0 International license.

The multiplex assay detected all HHVs correctly from infected cell lines without cross-amplification of other HHVs, human DNA, or near-full-length or full-length genomes of parvovirus B19 (B19V) or the polyomaviruses BK virus (BKPyV), JC virus (JCPyV), and Merkel cell virus (MCPyV). All the no-template water controls remained negative throughout the PCR analyses.

### Repeatability and reproducibility.

We tested the intra-assay and interassay variations in three separate qPCR runs using five replicates of the individual HHV plasmids (10^6^ to 10^1^ copies/μl) and plasmid mixes pMIXI (HSV-1 and -2 and VZV), pMIXII (EBV, HCMV, and KSHV), and pMIXIII (HHV-6A, -6B, and -7).

The assay showed excellent short-term repeatability and long-term reproducibility in both singleplex and multiplex formats as well as with pMIXI to -III. The highest standard deviations in quantification cycle (*C_q_*) values (intra-assay) and coefficients of variation between runs (interassay) were seen at the lowest template copies (Tables 1 and 2).

**TABLE 1:** Intra-assay variation

Virus and format	Mean *C_q_* value ± SD by no. of copies/μl						Efficiency (%)	*R*^2^	Slope	Intercept
	10^1^	10^2^	10^3^	10^4^	10^5^	10^6^				
HSV-1										
Singleplex	33.2 ± 0.4	30.3 ± 0.3	26.9 ± 0.1	23.4 ± 0.1	20.1 ± 0.1	16.9 ± 0.1	100.1	0.998	−3.32	39.1
Multiplex	33.2 ± 0.4	30.3 ± 0.3	26.8 ± 0	23.3 ± 0.1	20.1 ± 0	17.1 ± 0.2	101.7	0.997	−3.28	38.9
pMIXI	33.1 ± 0.1	30.5 ± 0.3	26.9 ± 0.1	23.5 ± 0.2	20.3 ± 0.2	16.8 ± 0.2	100.3	0.997	−3.32	39.1
HSV-2										
Singleplex	32.4 ± 0.7	29.2 ± 0.2	26.1 ± 0.3	22.6 ± 0.2	19.4 ± 0.4	16.3 ± 0.2	101	0.994	−3.3	38.3
Multiplex	32.4 ± 0.6	29.1 ± 0.2	25.8 ± 0.1	22.6 ± 0.2	19.2 ± 0.1	16.1 ± 0.1	101.7	0.997	−3.28	38.9
pMIXI	32.9 ± 0.6	29.4 ± 0.3	25.9 ± 0.1	22.5 ± 0.2	19.2 ± 0.1	16 ± 0.2	100.5	0.998	−3.31	38.2
VZV										
Singleplex	32.0 ± 0.4	28.9 ± 0.2	25.7 ± 0.2	22.1 ± 0.2	18.8 ± 0.1	15.8 ± 0.1	103.2	0.997	−3.25	37.5
Multiplex	32.5 ± 0.3	28.8 ± 0.2	25.5 ± 0.1	22.1 ± 0.2	18.9 ± 0.4	15.8 ± 0.2	100.9	0.997	−3.3	37.8
pMIXI	32.1 ± 0.4	28.7 ± 0.2	25 ± 0.3	22 ± 0.1	18.8 ± 0.1	15.6 ± 0.2	100.7	0.997	−3.31	37.6
EBV										
Singleplex	32.6 ± 0.4	29.0 ± 0.5	25.6 ± 0.1	22.3 ± 0.3	18.9 ± 0.1	15.7 ± 0.1	97.8	0.998	−3.37	38.2
Multiplex	32.6 ± 0.5	29.0 ± 0.2	25.7 ± 0.1	22.1 ± 0.1	18.9 ± 0.2	15.6 ± 0.1	99.5	0.998	−3.33	37.9
pMIXII	32.6 ± 0.6	29.1 ± 0.2	25.5 ± 0.1	22 ± 0.2	18.8 ± 0.2	15.5 ± 0.2	98.6	0.999	−3.36	37.9
HCMV										
Singleplex	33.9 ± 0.4	30.3 ± 0.3	26.9 ± 0.1	23.7 ± 0.1	20.4 ± 0.2	16.9 ± 0.1	98.2	0.997	−3.37	39.5
Multiplex	33.5 ± 0.7	30.4 ± 0.4	26.9 ± 0.2	23.6 ± 0.2	20.3 ± 0.1	16.8 ± 0.1	98.4	0.994	−3.36	39.4
pMIXII	33.6 ± 0.4	30.6 ± 0.3	27.0 ± 0.3	23.7 ± 0.2	20.4 ± 0.1	17.0 ± 0.1	99.7	0.996	−3.33	39.3
KSHV										
Singleplex	33.6 ± 0.8	29.4 ± 0.4	25.8 ± 0.1	22.6 ± 0.2	19.3 ± 0.1	16.1 ± 0.1	95.9	0.996	−3.42	38.8
Multiplex	32.6 ± 0.8	29.3 ± 0.5	26.1 ± 0.2	22.6 ± 0.2	19.1 ± 0.1	16.0 ± 0.1	98.9	0.992	−3.35	38.3
pMIXII	32.9 ± 0.7	28.9 ± 0.4	25.7 ± 0.2	22.4 ± 0.1	19.0 ± 0.2	15.5 ± 0.2	97.2	0.996	−3.39	38.3
HHV-6A										
Singleplex	32.4 ± 0.6	29.5 ± 0.3	26.1 ± 0.2	22.7 ± 0.1	19.2 ± 0.1	16.3 ± 0.1	100.6	0.995	−3.31	38.3
Multiplex	33.0 ± 0.4	29.5 ± 0.4	25.8 ± 0.1	22.5 ± 0.1	19.4 ± 0.2	16.6 ± 0.1	100.2	0.995	−3.32	38.4
pMIXIII	33.3 ± 0.6	29.6 ± 0.3	26.1 ± 0.3	22.9 ± 0.2	19.6 ± 0.2	16.1 ± 0.1	98.2	0.995	−3.37	38.7
HHV-6B										
Singleplex	31.6 ± 0.9	28.7 ± 0.4	25.3 ± 0.1	21.8 ± 0.2	18.6 ± 0.1	15.6 ± 0.1	100.3	0.992	−3.32	37.6
Multiplex	31.8 ± 0.5	28.6 ± 0.2	25.3 ± 0.1	22.0 ± 0.2	18.8 ± 0.2	15.7 ± 0.1	103.8	0.998	−3.23	37.4
pMIXIII	31.5 ± 0.5	29.1 ± 0.3	25.6 ± 0.2	22.2 ± 0.1	19 ± 0.2	15.8 ± 0.1	101.9	0.998	−3.28	37.7
HHV-7										
Singleplex	31.5 ± 0.7	28.1 ± 0.4	25 ± 0.1	21.6 ± 0.2	18.1 ± 0.1	15.0 ± 0.2	100.7	0.995	−3.31	37.01
Multiplex	31.6 ± 0.7	28.2 ± 0.4	24.9 ± 0	21.5 ± 0.1	18.2 ± 0.1	15.2 ± 0.1	102.7	0.993	−3.26	36.9
pMIXIII	32.2 ± 0.7	28.3 ± 0.1	25.0 ± 0.1	21.5 ± 0.2	18.4 ± 0.1	15.1 ± 0.1	98.2	0.994	−3.37	37.5

**TABLE 2:** Interassay variation

Virus and format	Coefficient of variation (%) by no. of copies/μl					
	10^1^	10^2^	10^3^	10^4^	10^5^	10^6^
HSV-1						
Singleplex	12	21	8	4	6	7
Multiplex	16	20	15	7	3	4
pMIXI	7	12	7	4	1	10
HSV-2						
Singleplex	23	12	11	9	4	9
Multiplex	27	10	3	2	9	6
pMIXI	33	8	7	9	5	3
VZV						
Singleplex	14	6	11	4	2	5
Multiplex	27	9	6	8	2	9
pMIXI	13	16	13	2	8	7
EBV						
Singleplex	24	11	11	12	11	7
Multiplex	23	4	10	1	2	4
pMIXII	20	7	4	12	4	14
HCMV						
Singleplex	15	13	9	11	18	10
Multiplex	14	9	6	2	3	6
pMIXII	16	8	5	7	12	3
KSHV						
Singleplex	15	13	9	11	18	10
Multiplex	14	9	6	2	3	6
pMIXII	16	8	5	7	12	3
HHV-6A						
Singleplex	15	7	14	9	3	12
Multiplex	4	19	9	3	1	15
pMIXIII	20	7	7	6	2	0
HHV-6B						
Singleplex	27	22	13	4	3	6
Multiplex	28	13	3	12	3	11
pMIXIII	19	5	6	10	6	4
HHV-7						
Singleplex	19	10	11	9	8	3
Multiplex	25	4	9	10	2	8
pMIXIII	31	14	12	3	4	14

The method was linear in the range of 10^1^ to 10^6^ copies per μl, and the qPCR efficiencies were between 95.9% and 103.8% in all the experiments.

**FIG 1:**
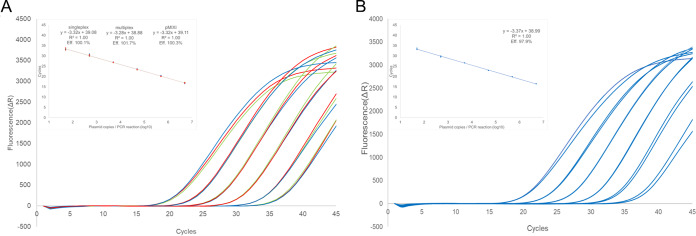
(A) Amplification and standard curves of HSV-1 plasmid dilutions from 10^6^ to 10^1^ copies/μl in singleplex format (red) or in multiplex format individually (green) or together with HSV-2 and VZV plasmids (pMIXI; blue). (B) pMIXI dilution series spiked with 500 ng/reaction of human DNA (HaCaT cells). The *y* axis represents baseline-corrected fluorescence signal (amplification curve) or cycles (standard curve). The *x* axis represents cycle number (amplification curve) or plasmid copies in the reaction (log_10_) (standard curve). Analogous illustrations for other herpesviruses can be found in Fig. S4.

The HHV plasmid dilutions spiked with 500 ng of human DNA (HaCaT cells) showed equal linearity to pure HHV plasmids (Fig. 1; see also Fig. S4 in the supplemental material).

10.1128/mSphere.00265-20.6 FIG S4 Amplification and standard curves in 10^6^ to 10^1^ copies/μl of plasmid dilutions of HSV-2 (A and B), VZV (C and D), EBV (E and F), HCMV (G and H), KSHV (I and J), HHV-6A (K and L), HHV-6B (M and N), and HHV-7 (O and P). On the left are represented plasmids in singleplex (red) and multiplex formats, both individually (green) or in pMIXI-III (blue). On the right are pMIXI-III dilution series spiked with 500 ng/reaction of human DNA (HaCaT cells). The *y* axis represents baseline-corrected fluorescence signal (amplification curve) or cycles (standard curve). The *x* axis represents cycle number (amplification curve) or plasmid copies in reaction(log_10_) (standard curve). Download FIG S4, PDF file, 2.3 MB. Copyright © 2020 Pyöriä et al. 2020 Pyöriä et al. This content is distributed under the terms of the Creative Commons Attribution 4.0 International license.

### Comparison to prequantified reference samples.

We correlated the quantification of HERQ-9 to several prequantified reference materials. The results are summarized in Table 3.

**TABLE 3:** Prequantified reference material

Virus	Sample type	Quantification method	Strain(s)	GenBank accession no.	No. of copies/μl		Conversion factor	Difference between quantifications (log_10_)
					Reference	HERQ-9		
HSV-1	DNA from purified nucleocapsids	Spectrophotometer	HSV-H1211, HSV-H1215	MH999843, MH999846	4.3 × 10^9^, 6.1 × 10^9^	4.2 × 10^9^, 6.1 × 10^9^	0.99	<0.02
HSV-2	DNA from purified nucleocapsids	Spectrophotometer	HSV2-H12211, HSV2-H1526	KY922725, KY922724	1.9 × 10^9^, 3.8 × 10^9^	9.1 × 10^8^, 1.9 × 10^9^	0.49	0.31
VZV	Quantified DNA, ATCC	ddPCR^*a*^	Ellen	JQ972913.1	5.6 × 10^5^	1.8 × 10^6^	3.2	0.51
EBV	WHO international standard	qPCR (NIBSC code 09/260) (57)	B95-8	NC_007605	1.0 × 10^4^	1.0 × 10^4^	1.0	<0.02
HCMV	WHO international standard	qPCR (NIBSC code 09/162) (58)	Merlin	GU179001.1	1.0 × 10^4^	3.2 × 10^4^	3.2	0.51
KSHV	Genome in BAC	Spectrophotometer	BAC16 JSC-1	MK208323.1	2.2 × 10^9^	5.0 × 10^9^	2.3	0.36
HHV-6A	Spiked serum, HHV-6 Foundation	qPCR (59)	GS	KC465951.1	1.6 × 10^4^ to 1.6 × 10^0^	2.1 × 10^4^ to 1.1 × 10^0^	1.1	0.03
HHV-6B	WHO international standard	qPCR (NIBSC code 15/266) (59)	Z29	AF157706.1	1.1 × 10^5^	1.8 × 10^5^	1.6	0.20
HHV-7	Spiked serum, HHV-6 Foundation	HHV-7 qPCR kit (PCRmax)	JI	U43400.1	1.7 × 10^2^ to 2.4 × 10^−1^	1.1 × 10^3^ to 2.4 × 10^0^	6.8	0.83

a ddPCR, droplet digital PCR.

DNA from each of the two strains of HSV-1 and HSV-2 had spectrophotometrically estimated quantities of 4.3 × 10^9^ and 6.1 × 10^9^ copies/μl and 1.9 × 10^9^ and 3.8 × 10^9^ copies/μl, respectively. The calculated loads by our multiplex qPCR were 4.2 × 10^9^ and 6.1 × 10^9^ copies/μl for HSV-1 and 9.1 × 10^8^ and 1.9 × 10^9^ copies/μl for HSV-2. The conversion factors were hence 0.99 (HSV-1) and 0.49 (HSV-2).

A VZV DNA extract (ATCC) containing 5.6 × 10^5^ copies/μl was calculated to have 1.8 × 10^6^ copies/μl by HERQ-9, with a conversion factor of 3.2.

EBV, HCMV, and HHV-6B WHO international standards contained 1.0 × 10^4^, 1.0 × 10^4^, and 1.1 × 10^5^ IU/μl, respectively. The calculated copies by HERQ-9 were 1.0 × 10^4^ copies/μl for EBV, 3.2 × 10^4^ copies/μl for HCMV, and 1.8 × 10^5^ copies/μl for HHV-6B. Hence, the conversion factors (copies/international unit) were 1.0, 3.2, and 1.6, respectively.

Spectrophotometrically estimated copies of KSHV DNA were 2.2 × 10^9^ copies/μl and calculated to be 5.0 × 10^9^ copies/μl with our assay, yielding a conversion factor of 2.3.

**FIG 2:**
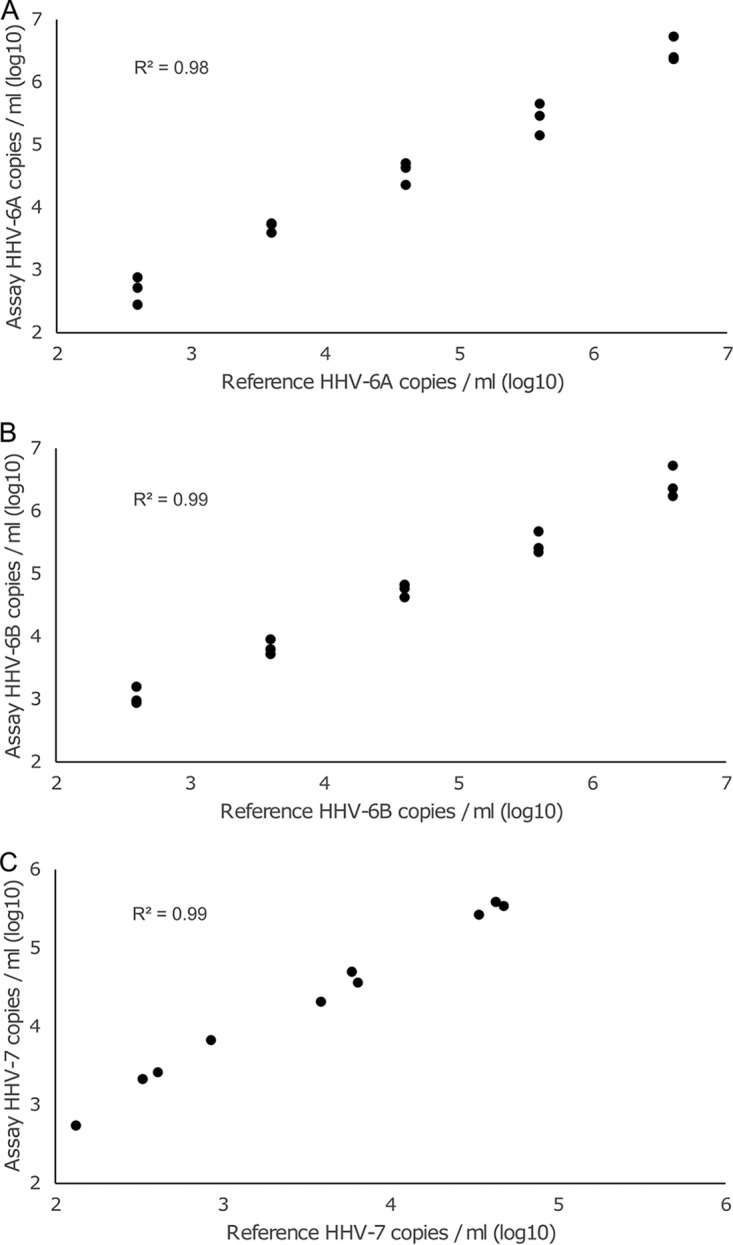
Reference copy numbers of serum samples spiked with HHV-6A (A), HHV-6B (B), and HHV-7 (C) plotted against copy numbers quantified by HERQ-9. Viral DNA copy numbers are presented per milliliter of serum (log_10_ transformed). *R*^2^, coefficient of determination between the copy numbers.

HERQ-9 showed good correlation with HHV-6A, HHV-6B, and HHV-7 spiked in sera (Fig. 2). The conversion factors were 1.1, 1.3, and 6.8, respectively.

### Analysis of clinical samples.

We tested several types of clinical samples and compared the positive and negative agreements against reference methods. A summary of the results is presented in Table 4.

**TABLE 4:** Clinical samples and other sample material

Virus	Clinical samples					Other sample material		
	Sample type	*n*	Reference method(s)	Positive agreement	Negative agreement	Type	Strain(s)	GenBank accession no.
HSV-1	Mucocutaneous swab	92	Rapid viral culture immunoperoxidase, qPCR (4, 31, 53)	42/42 (100%)	49/50 (98%)	Infected cell cultures	Strain F	KM222724.1
HSV-2	Mucocutaneous swab		Rapid viral culture immunoperoxidase, qPCR (4, 31, 53)	34/34 (100%)	58/58 (100%)	Infected cell cultures	Strain G	KP143740.1
VZV	Mucocutaneous swab	27	EIA, qPCR (4, 31, 61)	20/20 (100%)	14/15 (93.3%)	Infected cell cultures	Ellen	JQ972913.1
	CSF	8						
EBV	Plasma	46	GeneProof EBV PCR kit	14/14 (100%); 7/9^*a*^	21/23 (91.3%)	Raji cells	Raji	KF717093.1
HCMV	Plasma	48	GeneProof CMV PCR kit	19/19 (100%); 4/5^*a*^	22/24 (91.7%)	Infected cell cultures	AD169	FJ527563.1
KSHV						Infected cell cultures	JSC-1 clone BAC16	GQ994935.1
HHV-6A						Infected cell cultures	GS	KC465951.1
HHV-6B						Spiked serum, infected cell cultures	Z29	AF157706.1
HHV-7						Infected cultures	JI	U43400.1

a Positive agreement with samples reported borderline with reference assay.

**FIG 3:**
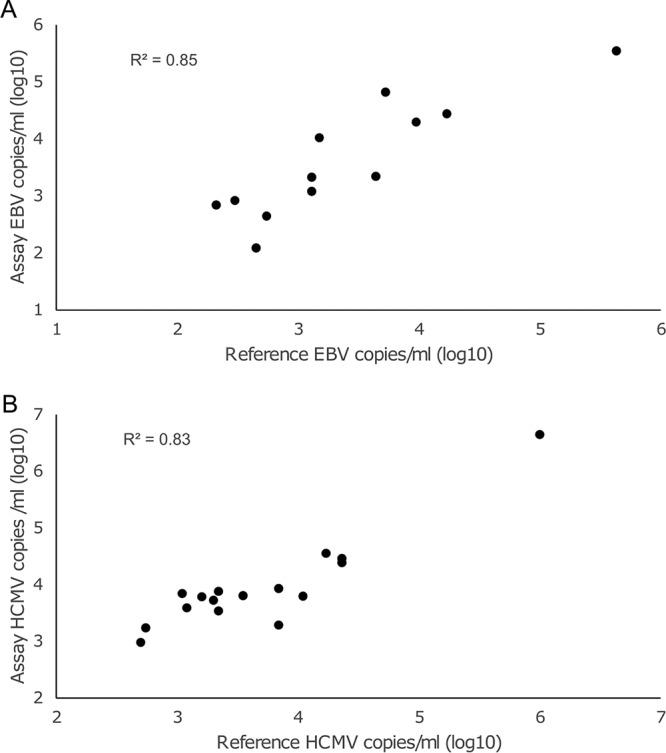
Comparison of EBV (A) and HCMV (B) quantities (copies/milliliter of plasma in log_10_) as determined by HERQ-9 and GeneProof EBV and CMV PCR kit reference assays.

**(i) Plasma.** We tested 60 plasma samples, previously studied for EBV and/or HCMV at Turku University Hospital. Altogether, 13/13 and 16/16 plasma samples positive for EBV and HCMV by a reference qPCR, respectively, were positive by HERQ-9, with good correlation in viral loads (Fig. 3). Seven out of nine samples reported as borderline for EBV by the clinical laboratory (50 to 200 copies/ml of plasma) were positive in the new assay, as were four of five HCMV-borderline samples (50 to 200 copies/ml of plasma). Furthermore, the new qPCR found additional samples positive for EBV (*n* = 3) and HCMV (*n* = 5) that in the hospital laboratory had been tested only for one of the viruses. These samples were reanalyzed with a reference qPCR confirming one EBV and three HCMV positivities.

**FIG 4:**
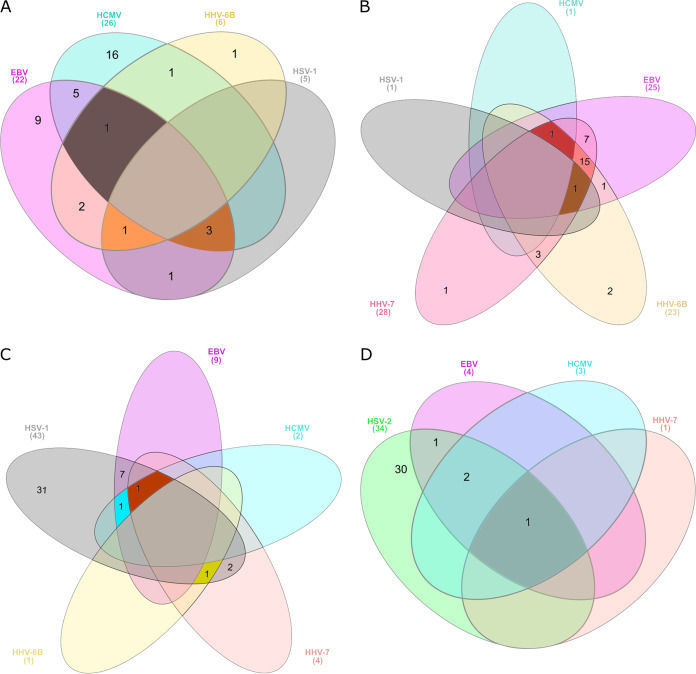
Venn diagrams representing HHV co-occurrence in 60 plasma samples (A), 35 palatine tonsils (B), 43 HSV-1-positive mucocutaneous swab samples (C), and 34 HSV-2-positive mucocutaneous swab samples (D). *n*, number of positive cases.

Of the 60 plasma samples studied with HERQ-9, 6 were positive for HHV-6B (median, 2.7 × 10^2^ copies/ml of plasma; range, 1.7 × 10^2^ to 5.2 × 10^3^), 1 was positive for HHV-6A (6.3 × 10^2^ copies/ml of plasma), and 5 were positive for HSV-1 (median, 1.2 × 10^4^ copies/ml of plasma; range, 8.0 × 10^2^ to 5.1 × 10^4^). The co-occurrence of HHVs in plasma was seen for 14 patients, of whom 3 had EBV/HCMV/HSV-1, 1 had EBV/HCMV/HHV-6B, 1 had EBV/HHV-6B/HSV-1, 5 had EBV/HCMV, 2 had EBV/HHV-6B, 1 had HCMV/HHV-6B, and 1 had EBV/HSV-1 (Fig. 4A).

### (ii) Mucocutaneous swabs.

We tested 114 mucocutaneous swab samples previously investigated for HSV-1 and -2 or VZV at Turku University Hospital.

HERQ-9 identified correctly all the mucocutaneous swab samples that had tested positive by rapid viral culture for HSV-1 (*n* = 35; median, 5.6 × 10^7^; range, 1.4 × 10^5^ to 8.2 × 10^9^ copies/ml of collection medium) and HSV-2 (*n* = 30; median, 3.3 × 10^7^; range, 2.9 × 10^5^ to 3.5 × 10^8^ copies/ml of collection medium). In contrast, the 15 culture-negative controls showed no amplification for HSV-1 or HSV-2. However, two of these negative samples were positive instead for VZV, at 2.0 × 10^7^ and 3.2 × 10^4^ copies/ml of collection medium, and were confirmed to be VZV DNA positive with a control PCR (4, 31). In addition, 5/5 HSV-1-positive and 4/4 HSV-2-positive DNA extracts previously tested by a reference PCR (4, 31) were also positive by HERQ-9.

All the VZV samples positive (*n* = 15) by enzyme immunoassay (EIA) were positive by the new assay, at a median quantity of 4.5 × 10^7^ copies/ml of collection medium (range, 8.0 × 10^6^ to 2.7 × 10^9^). On the other hand, among 10 VZV antigen-negative samples, 2 contained VZV DNA at 3.6 × 10^6^ and 4.7 × 10^3^ copies/ml of collection medium. Of these, the former was confirmed to be VZV DNA positive by the reference PCR. Incidentally, among the remaining eight samples negative for the VZV antigen, three showed positivity for HSV-1 instead, at loads of 7.7 × 10^7^, 7.2 × 10^7^, and 6.3 × 10^1^ copies/ml of collection medium. Only the two samples with the highest copy numbers were confirmed to be positive for HSV-1 DNA by the control PCR.

Moreover, we codetected other HHVs in these mucocutaneous swabs (Fig. 4C and D). Of the HSV-1-positive samples, 20.9% were also positive for EBV DNA, 4.7% for HCMV DNA, 9.3% for HHV-7 DNA, and 2.3% for HHV-6B DNA. Among the HSV-2-positive swabs, 5.3% were also positive for EBV, 8.8% for HCMV, and 2.9% for HHV-7. Of 19 VZV-positive swabs, 1 was positive for EBV-DNA (5.3%). Of all the 114 swabs, 2 were quadruply positive (HSV-1/EBV/HCMV/HHV-7 and HSV-2/EBV/HCMV/HHV-7) and 4 were triply positive (HSV-1/EBV/HCMV, HSV-1/HHV-6B/HHV-7, and two HSV-2/EBV/HCMV). The copy numbers of the other codetected HHVs (generally log_2_ to log_3_ copies/ml of collection medium) were always lower than those for HSV-1, HSV-2, or VZV. However, a few samples had log_5_ to log_6_ copies/ml of EBV DNA. Of all the mucocutaneous swab samples negative for HSV-1, HSV-2, and VZV (*n* = 18), one (5.6%) tested positive for EBV DNA.

### (iii) CSF.

Eight cerebrospinal fluid (CSF) samples were analyzed with the pan-herpes multiplex assay. Two were positive for VZV (3.23 × 10^3^ and 1.64 × 10^5^ copies/ml of CSF), in concordance with the hospital laboratory reference PCR (4, 31).

### HHV prevalences in tonsillar tissue.

**FIG 5:**
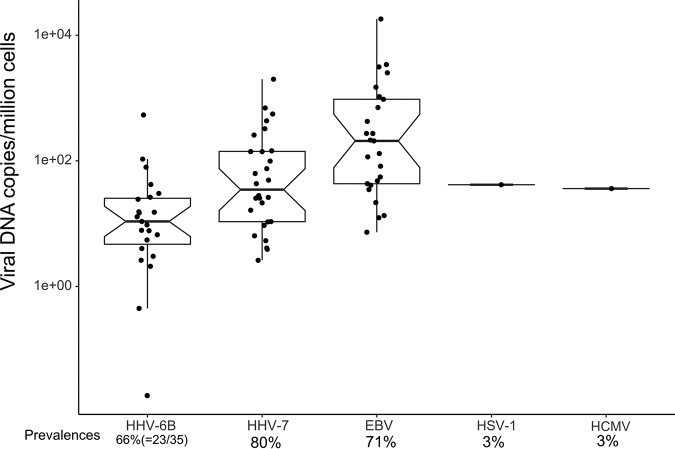
Prevalences and copy numbers per million cells of HHV-6B, HHV-7, EBV, HSV-1, and HCMV in tonsillar tissues. Notches represent interquartile ranges (IQRs) of the samples and whiskers the range ±1.5 IQRs from the upper and lower quartiles.

The HHV DNA prevalences in 35 tonsillar tissue samples were 80% for HHV-7, 71% for EBV, 66% for HHV-6B, 3% for HSV-1, and 3% for HCMV. No HSV-2, VZV, or KSHV was found (Fig. 5). Of these samples, 2 were positive for four HHVs, 17 for three, and 10 for two, while four tonsils were negative for all the HHVs (Fig. 4B). The median viral loads (copies/million cells) were highest for EBV (2.1 × 10^2^; interquartile range [IQR], 9.6 × 10^2^), followed by HHV-7 (3.6 × 10^1^; IQR, 1.3 × 10^2^) and HHV-6B (1.1 × 10^1^; IQR, 2.2 × 10^1^).

### Coquantification of mixed high- and low-abundance targets.

We tested uneven copies of whole HHV genomes in the same reaction (range, 4.5 × 10^0^ to 1.1 × 10^6^ copies/μl) and found that all the viruses were correctly differentiated and accurately quantified by HERQ-9 (Pearson correlation coefficient [*r*] = 0.996, *P* < 0.01). Higher coefficients of variation were seen at lower viral copy numbers (Table 5).

**TABLE 5:** Three viral genomes intermixed into the same reaction^*a*^

Virus mix	Estimated viral genome copies/μl			Measured viral genome copies/μl		
	HSV-1	HSV-2	VZV	HSV-1	HSV-2	VZV
1	1.1 × 10^6^	5.6 × 10^3^	2.8 × 10^2^	1.2 × 10^6^ (6)	1.0 × 10^4^ (42)	1.9 × 10^2^ (28)
2	1.3 × 10^4^	5.9 × 10^4^	3.4 × 10^1^	1.3 × 10^4^ (1)	6.3 × 10^4^ (4)	1.4 × 10^1^ (57)
3	1.2 × 10^3^	6.2 × 10^3^	4.2 × 10^4^	1.1 × 10^3^ (9)	5.1 × 10^3^ (13)	2.7 × 10^4^ (29)
4	1.2 × 10^2^	4.5 × 10^1^	4.2 × 10^3^	1.1 × 10^2^ (9)	7.2 × 10^1^ (33)	3.3 × 10^3^ (15)
5	1.1 × 10^5^	4.6 × 10^1^	3.6 × 10^4^	1.1 × 10^5^ (2)	8.9 × 10^1^ (45)	3.0 × 10^4^ (13)
6	1.2 × 10^2^	5.9 × 10^4^	4.2 × 10^4^	1.6 × 10^2^ (18)	4.9 × 10^4^ (13)	4.2 × 10^4^ (1)
	EBV	HCMV	KSHV	EBV	HCMV	KSHV
7	1.1 × 10^5^	1.5 × 10^3^	5.4 × 10^3^	1.2 × 10^5^ (5)	2.6 × 10^3^ (37)	6.7 × 10^3^ (15)
8	1.1 × 10^4^	1.7 × 10^5^	4.9 × 10^5^	1.3 × 10^4^ (12)	1.8 × 10^5^ (4)	6.4 × 10^5^ (20)
9	1.2 × 10^3^	1.8 × 10^4^	5.7 × 10^4^	1.4 × 10^3^ (10)	1.5 × 10^4^ (11)	6.9 × 10^4^ (14)
10	7.1 × 10^1^	1.3 × 10^2^	6.0 × 10^5^	1.1 × 10^2^ (31)	1.9 × 10^2^ (26)	6.2 × 10^5^ (2)
11	1.1 × 10^5^	1.7 × 10^5^	4.1 × 10^2^	1.2 × 10^5^ (6)	2.0 × 10^5^ (12)	9.7 × 10^2^ (57)
12	9.6 × 10^1^	1.8 × 10^4^	5.7 × 10^4^	1.3 × 10^2^ (22)	1.5 × 10^4^ (14)	5.4 × 10^4^ (3)
	HHV6-A	HHV6-B	HHV-7	HHV6-A	HHV6-B	HHV-7
13	1.9 × 10^3^	6.2 × 10^3^	1.0 × 10^2^	1.7 × 10^3^ (9)	7.1 × 10^3^ (9)	1.3 × 10^2^ (24)
14	1.9 × 10^3^	6.1 × 10^2^	1.0 × 10^2^	1.7 × 10^3^ (10)	4.6 × 10^2^ (18)	1.1 × 10^2^ (10)
15	9.3 × 10^1^	6.1 × 10^3^	5.2 × 10^1^	1.2 × 10^2^ (21)	5.6 × 10^3^ (6)	3.6 × 10^1^ (25)
16	1.3 × 10^3^	6.1 × 10^1^	5.2 × 10^1^	1.4 × 10^3^ (9)	1.2 × 10^2^ (47)	3.6 × 10^1^ (26)
17	4.5 × 10^0^	7.9 × 10^1^	1.0 × 10^2^	1.1 × 10^1^ (58)	5.2 × 10^1^ (30)	1.1 × 10^2^ (4)
18	1.3 × 10^3^	6.1 × 10^3^	5.2 × 10^1^	1.9 × 10^3^ (27)	1.1 × 10^4^ (40)	4.9 × 10^1^ (4)

a On the left are the estimated genome copies per dilution and on the right the copy numbers per microliter of DNA extract quantified by HERQ-9. In parentheses are the coefficient of variations (percent) between the estimated and measured copy numbers. Estimated and measured viral genome copies showed significant correlation (Pearson’s *r* = 0.996; *P* < 0.01).

## DISCUSSION

Our newly developed pan-herpes multiplex-qPCR assay, HERQ-9, stands out for its ability to differentiate and quantify the genomes of all nine human herpesviruses.

HERQ-9 was designed on three distinct triplex-qPCRs to meet, on the one hand, the clinical needs and, on the other, the technical constraints inherent to PCR multiplexing. Indeed, the capacity to codetect several targets is restricted by the spectral overlap of different fluorophores as well as the number of channels in the qPCR instrument (maximum of six) (32). Moreover, a greater number of targets can increase cross-reactions between primers and probes, hampering assay performance.

The new multiplex assay performed remarkably well on cell, plasma, and CSF samples and mucocutaneous swabs as well as on palatine tonsils. HHV genoprevalences in this lymphoid organ have been reported to be highest for EBV (20.4 to 88.8%), followed by HHV-7 (71.4%), HHV-6B (50.7%), HSV-1 (1.8 to 6.3%), and HCMV (0 to 5.4%) (33–35), in line with our results. In addition, we frequently codetected several HHVs in the same tonsil, a phenomenon only previously reported by Berger et al. for young children (36).

HERQ-9 had good agreement with reference materials. The observed dissimilarities were likely to be related to the types and sensitivities of different methodologies (e.g., viral culture, EIA, and spectrophotometry), sample processing (e.g., DNA extraction methods), and the design of the PCR methods compared. Regarding the last item, the primer and probe design, amplicon size, target gene and its polymorphisms, reagents, and standards can all account for disagreements between qPCRs (37–39). In fact, these discrepancies have urged the introduction of WHO international standards for EBV, HCMV, and HHV-6B to increase the commutability between assays (39–42). However, this has had only relative value since, even after standardization, the interlaboratory variabilities continue to be high (up to 1.5 log_10_ IU/ml, on average) (39, 42).

Our findings emphasize the importance of multiplexing for comprehensive diagnosis and clinical management. Indeed, we identified additional HHVs in clinical samples that had been tested only for a single pathogen, encountering several unforeseen HSV-1 or VZV findings in mucocutaneous swabs, as well as EBV-HCMV coreactivations in plasma of immunodeficient patients. In addition, we codetected other HHVs in plasma in several combinations (EBV/HSV-1, EBV/HHV-6B, HCMV/HHV-6B, EBV/HCMV/HSV-1, EBV/HCMV/HHV-6B, and EBV/HHV-6B/HSV-1). These coincidental discoveries, also noted by others (4, 19, 43), may have a significant impact on risk assessment and prognosis. Indeed, HHVs are thought to individually or synergistically contribute to viral syndromes (5, 8, 44), organ rejection (19), or the development of cancer (45, 46).

Moreover, we found other HHVs besides HSV-1, HSV-2, or VZV in mucocutaneous lesions (up to four in the same sample). The most common were EBV, HCMV, and HHV-7, whose low viral loads were likely to represent skin virome (47) or latency in mobilized leukocytes (48). Yet in a few samples, EBV DNA levels approached those of HSV-1 or HSV-2, suggestive of *in situ* coreactivation or coinfection. Our detection of both EBV and HSV-1 in mucocutaneous lesions and plasma supports an interplay between these two viruses, as has been shown *in vitro* by Wu et al. (49). To the best of our knowledge, we are the first to report on HHV co-occurrences in classical herpetic lesions.

In conclusion, we demonstrated that HERQ-9 is suitable for the diagnosis of a plethora of herpesvirus-related diseases. Besides its significance for clinical management, the high sensitivity and specificity of this method will be of particular value for studies of the human virome generally dealing with minute quantities of persisting HHVs.

## MATERIALS AND METHODS

### Plasmids.

Sequences of the reference strain (90 to 336 bp), including the corresponding qPCR amplicon, were inserted into a pIDTSmart backbone (Integrated DNA Technologies). The plasmids (pHSV-1, pHSV-2, pVZV, pHHV-6A, pHHV-6B, pHHV-7, pEBV, pHCMV, and pKSHV) were transformed into Escherichia coli, extracted (see “DNA extraction” below), and confirmed with restriction analysis to contain the correct insert (for whole insert sequences, see Text S1 in the supplemental material). The concentrations were measured spectrophotometrically, and the plasmids (diluted serially from 10^6^ to 10^1^ copies/μl in 10 mM Tris-EDTA [TE] buffer) were stored at –80°C. In addition to single-plasmid analysis, triple-plasmid combinations were made: pHSV-1, pHSV-2, and pVZV (pMIXI); pEBV, pHCMV, and pKSHV (pMIXII); and pHHV-6A, pHHV-6B, and pHHV-7 (pMIXIII).

10.1128/mSphere.00265-20.1 TEXT S1 Sequences of reference strains in the plasmids. Download Text S1, DOCX file, 0.01 MB. Copyright © 2020 Pyöriä et al. 2020 Pyöriä et al. This content is distributed under the terms of the Creative Commons Attribution 4.0 International license.

Plasmids containing the full-length or near-full-length genome of parvovirus B19 genotype 1 (50) and polyomaviruses BKPyV (NC_001538) and JCPyV (NC_001699) (generous gifts from Eeva Auvinen) and MCPyV (inserted in vector backbone pJ241, a gift from Patrick Moore [51]; Addgene plasmid 32059) were used to test nonspecific amplification.

### Infected cell cultures.

Primers and probes were initially tested using viral DNA extracted from virus-infected cell cultures. HSV-1 (strain F), HSV-2 (strain G), and VZV (strain Ellen) were propagated in HaCat cells, human foreskin fibroblasts, and Vero cells; EBV was propagated in Raji cells, HCMV (strain AD169) in human lung fibroblasts (MRC-5), HHV-6A (strain GS) in HSB-2 cells, HHV-6B (strain Z29) in MOLT-3 cells, HHV-7 (strain JI) in SupT-1 cells, and KSHV (strain rKSHV.219) in latent and lytic iSLK.219 cells (52).

Uninfected HaCaT cells were used for human DNA spiking experiments.

### Prequantified reference material.

All the reference materials are presented in Tables 3 and 4.

**(i) Cell-free viral nucleocapsids.** HSV-1 and HSV-2 nucleocapsids were isolated from pseudonymized dermal or mucosal lesion samples at the virus diagnostic unit of Turku University Hospital. The viruses were initially typed by a rapid viral culture immunoperoxidase assay (53) and confirmed by HSV type-specific gD (US6) gene-based PCR (54). For viral nucleocapsid DNA preparations, low-passage-number stocks were generated in Vero cells (African green monkey kidney; ATCC), and the viral genomic DNA was prepared as described previously (55, 56) (see Text S2 in the supplemental material for a more detailed description). Two strains of HSV-1 (HSV-H1211 and HSV-H1215) and HSV-2 (HSV2-H12211 and HSV2-H1526) were prepared and the viral copies determined spectrophotometrically to be used as reference standards in dilutions of 1:10,000 and 1:100,000.

10.1128/mSphere.00265-20.2 TEXT S2 Preparation protocol for HSV-1 and HSV-2 nucleocapsids. Download Text S2, DOCX file, 0.01 MB. Copyright © 2020 Pyöriä et al. 2020 Pyöriä et al. This content is distributed under the terms of the Creative Commons Attribution 4.0 International license.

**(ii) WHO international standards.** WHO international standards for EBV, HCMV, and HHV-6B (NIBSC codes 09/260, 09/162, and 15/266, respectively) (57–59) were tested undiluted and in dilutions of 1:10 and 1:100 with HERQ-9, to obtain conversion factors (viral DNA copies/international units).

**(iii) KSHV genome in bacterial artificial chromosome (BAC).** KSHV-BAC16 DNA (a generous gift from Carolina Arias, University of California, Santa Barbara [UCSB], CA), derived from the KSHV strain of primary effusion lymphoma (PEL) cell line JSC-1, was purified from E. coli (60). The viral copy numbers were estimated spectrophotometrically. This reference was analyzed in dilutions of 1:10,000, 1:100,000, and 1:1,000,000 with the multiplex assay.

**(iv) Spiked sera.** Serum samples spiked with HHV-6A (*n* = 15) or HHV-6B (*n* = 15) (4 × 10^6^ to 4 × 10^2^ copies/ml of serum) and HHV-7 (*n* = 12) were obtained from the HHV-6 Foundation (Santa Barbara, CA). Reference copy numbers given by the providing institute were used for HHV-6A and HHV-6B (59), while the HHV-7 DNA was quantified with a commercial HHV-7 qPCR kit (PCRmax) in our laboratory.

**(v) ATCC standard.** Quantitative genomic DNA of VZV (ATCC VR1367DQ) was analyzed with HERQ-9 undiluted and at 1:10 and 1:100 dilutions.

### Clinical samples.

All the clinical HHV samples were collected at the virus diagnostic unit of Turku University Hospital, and details are presented in Table 4. These included 80 mucocutaneous swab samples, of which 35 were positive for HSV-1 and 30 for HSV-2 by rapid viral culture immunoperoxidase assay (53), 25 mucocutaneous swab samples, of which 15 were positive for VZV by antigen enzyme immunoassay (61), 5 HSV-1 and 4 HSV-2 PCR-positive DNA extracts from mucocutaneous swab samples tested by a reference PCR (4, 31), and 8 CSF samples, of which 2 were VZV positive by a control PCR (4, 31).

In addition, 60 plasma samples were investigated, of which 17 had been studied only for EBV (GeneProof EBV PCR kit), 17 only for HCMV (GeneProof CMV PCR kit), and 26 for both. Of these, 13 and 16 samples were reported as EBV and HCMV positive (>200 copies/ml of plasma), respectively, while 9 and 5 had borderline copy numbers (50 to 200 copies/ml of plasma).

### Tonsillar tissues.

Altogether, 35 mechanically homogenized tonsillar tissues were screened for the nine HHVs. The patients were 2 to 69 years of age (mean, 26), with eight <12 years (48). The viral loads were normalized per 10^6^ cells, determined with the human single-copy gene RNase P qPCR (48).

### Herpesvirus DNA mixes.

Mixtures of three viral genomes extracted from the previously mentioned infected cell lysates ([i] HSV-1, HSV-2, and VZV and [ii] EBV, HCMV, and KSHV) or spiked serum ([iii] HHV-6A, -6B, and -7) were tested at uneven quantities, ranging from 4.5 × 10^0^ to 1.1 × 10^6^ copies/μl of DNA extract.

### Primers and hydrolysis probes.

Primers and probes were designed for all HHVs, except for EBV (62). For each virus, several primer pairs were constructed *in silico*, in conserved genes (16, 18, 63, 64). Degenerate primers were designed for HCMV to cover polymorphisms in the target area. Moreover, for HHV-6A, two probes were custom-designed to contain six locked nucleic acids (LNA) each (for shorter probe length) and a single-nucleotide difference for specific binding to different strains (Table 6).

**TABLE 6:** Primers and probes

Virus	Oligonucleotide name	Concn (nM)	Sequence (5′–3′)^*a*^	Positions of the amplicon in the genome (length)	Target gene	Reference sequence (GenBank)
HSV-1	HSV-1 FWDLP1	300	GTTGAGCTAGCCAGCGA	93560–93683 (124 bp)	UL42	X14112.1
	HSV-1 REVLP1	300	CGTTAAGGACCTTGGTGAGC			
	HSV-1 probeLP1	250	FAM-CGCGAACTGACGAGCTTTGTG-BHQ1			
HSV-2	HSV-2 FWD-2-2	400	CACACCACACGACAACAA	46783–46872 (90 bp)	UL23	Z86099.2
	HSV-2 REVLP1	400	TAGTTCAAACACGGAAGCC			
	HSV-2 probeLP1	200	JOE-CGGCGATGACGGCAATAAA-BHQ1			
VZV	VZV FWDLP1	200	GCGCAAGGCTATTAGAGC	48283–48145 (139 bp)	ORF28	KU529566.1
	VZV REVLP1	200	ACATGGCAGAAATCCCTG			
	VZV probeLP1	150	TxRd-CGCATACCCGGAAGTTCTTCAGAT-BHQ2			
EBV	EBV FWD	200	CGGAAGCCCTCTGGACTTC	153036–152947 (90 bp)	BALF5	KF717093.1
	EBV REV	300	CCCTGTTTATCCGATGGAATG			
	EBV Probe	300	FAM-TGTACACGCACGAGAAATGCGCC-BHQ1			
HCMV	H5 FWD211	400	GTGYTCCGTGAATCGTTAC	80396–80329 (68 bp)	UL54	AB329634.1
	H5 rev 211	500	AGTCKACCTCGATATCACAAGTCG			
	H5 Probe 20	300	TxRd-ACCCTGCTGCCGCCAGT-BHQ2			
KSHV	HHV8 fwd 3.1	200	ATATACGGCGACACTGACTC	13603–13761 (159 bp)	ORF9	AP017458.1
	HHV8 REV 10	200	GAGCAGAAGGCACTTGAAG			
	H8 Probe 300	200	JOE-CGGAGGAGCTAGCGTCAATCA-BHQ1			
HHV-6A	HHV6A FWD1-3	500	CGGCCTCCAGAGTTGTAA	133969–133894 (76 bp)	U90	KP257584.1
	HHV6A REV 10	500	TGTCCCTTCAACTACTGAATC			
	HHV6A LNA Probe A1	100	FAM-AC[+A]T[+G]TTGC[+T]A[+G]AAA[+G][+A]CT-BHQ1			
	HHV6A LNA Probe A2	100	FAM-AC[+A]T[+G]TTGC[+T]A[+C]AAA[+G][+A]CT-BHQ1			
HHV-6B	H6B FOTY1	300	TTTGACAGGAGTTGCTGAG	136176–136258 (83 bp)	U90	AB021506.1
	H6B ROTY 1	300	GGATTCAGGAAAAAGGTTCTAA			
	H6B PROBE MVP	200	JOE-AGGAAGCGTTTCGGTACACTTGGAG-BHQ1			
HHV-7	HHV7 1. FWD	400	CTCGCAGATTGCTTGTTG	88332–88490 (159 bp)	U57	AF037218.1
	HHV7 1. REV	400	GCATACACCAACCCTACTGTAA			
	H7 MOP PROBE	300	TxRd-TTAGGCATCACGTTGGCATTG-BHQ2			

a Nucleotides in brackets refer to locked nucleic acids.

The tendency of primers and probes to form secondary structures, primer dimers, and cross-dimers was evaluated with Multiple Primer Analyzer (Thermo Fisher Scientific), while the propensity of the amplicons to form secondary structures was checked with the Mfold web server. A BLAST search (65) was performed to confirm primer binding to each of the virus strains in the nucleotide collection database (NCBI).

Primer candidates designed *in silico* were tested at a 200 nM concentration with plasmid dilutions (see “Plasmids” above), human DNA (HaCaT; 500 ng/reaction), and nuclease-free water in a SYBR green format (Maxima SYBR green qPCR master mix; Thermo Fisher Scientific), followed by melting-curve analysis. Primer pairs showing the highest efficiency and sensitivity with no primer dimer formation were chosen for further testing with the hydrolysis probes. Concentrations of the primer pairs were optimized empirically with a matrix of reactions ranging from 100 nM to 600 nM. The probes were tested in a 100 nM to 400 nM range. The final primer and probe concentrations are presented in Table 6.

Primers and hydrolysis probes were purchased as high-performance liquid chromatography (HPLC) purified except for two degenerative primers (HCMV), which were cartridge purified (Sigma-Aldrich). For the triplex reactions, the probes were labeled with 6-carboxyfluorescein–black hole quencher 1 (FAM-BHQ1), 6-carboxy-4′,5′-dichloro-2′,7′-dimethoxyfluorescein (JOE)-BHQ1, and sulforhodamine 101 acid chloride-BHQ2 (TxRed).

### Quantitative PCR protocol.

Four commercial master mixes were pretested with HSV-1, HSV-2, and VZV plasmids and viral genomes, with special consideration given to the performance in the presence of human DNA and the coamplification of markedly low- and high-abundance targets. Consequently, TaqPath ProAmp multiplex master mix (Thermo Fisher Scientific) was chosen for the multiplex assay.

The qPCR thermal profile comprised initial denaturation at 95°C for 10 min followed by 45 cycles of 15 s at 95°C and 60 s at 60°C. The qPCRs contained 5 μl of template, 2× TaqPath ProAmp multiplex master mix, primers and probes (Table 6), and nuclease-free water in a final volume of 20 μl. Water was used as negative control in all the qPCR runs. The samples were run in duplicate in AriaMx real-time PCR system (Agilent) and analyzed with Aria real-time PCR software (v.1.3) provided by the manufacturer. The adaptive fluorescence baseline, efficiency, slope, *R*^2^ values, and intercept were calculated by the software. Background-based threshold was set for cycles 5 to 9 for the FAM and Texas Red dyes and 8 to 11 for the JOE dye.

Pretesting of primers in the SYBR green format consisted of the above-mentioned thermal profile, followed by a melting-curve analysis at 95°C for 60 s, 45°C for 30 s, and 95°C for 30 s. The melting-curve analysis was performed with a resolution of 0.5°C and soak time of 5 s.

### DNA extraction.

DNAs from plasma, mucocutaneous swabs, CSF, and WHO international standards were extracted from 200 μl of starting material with the QIAamp DNA blood minikit (Qiagen), DNAs from cells and virus-infected cell lines were extracted with the QIAamp DNA minikit (Qiagen), DNA from KSHV BAC was extracted with the NucleoBond Xtra Midi EF kit (Macherey-Nagel), and transformed plasmids were extracted with GeneJET plasmid miniprep kit (Thermo Fisher Scientific), according to the manufacturers’ instructions. The final elution volumes were 100 μl (with the exception of 50 μl for plasma and 60 μl for CSF samples). In every extraction, at least two negative controls (phosphate-buffered saline) were included.

### Analytical sensitivity and specificity.

The analytical sensitivities were determined in singleplex and multiplex formats using eight replicates of each HHV plasmid template in 50, 25, 15, 10, 5, 3, and 1 copy per reaction. The proportion of positive results was fit into a generalized linear model using probit link function (MATLAB v.R2018b) to approximate the limit of detection (LOD_95_) for a given target.

The analytical specificities were evaluated by cross-testing (i) 10^7^ copies of viral genomic DNA extracted from infected cell lysates and plasmid constructs of each HHV and (ii) plasmids containing near full-length or full-length genomes of polyomaviruses BKPyV, JCPyV, and MCPyV and parvovirus B19 genotype 1. In addition, 1,000 ng of cellular DNA extracted from HaCaT cells and 500 ng from human foreskin fibroblasts were tested for nonspecific amplification of human DNA.

### Repeatability and reproducibility.

The intra-assay and interassay variations were calculated using three separate qPCR runs using five replicates of HHV plasmids (10^6^ to 10^1^ copies/μl) in the singleplex and multiplex formats, the latter both as single plasmids or in mixes pMIXI, pMIXII, and pMIXIII (10^6^ to 10^1^ copies/μl). Two of the replicates were used to generate a standard curve, and three were marked as unknowns. The standard deviations of the *C_q_* values of the five replicates were used as a measure of intra-assay variation. A coefficient of variation calculated from the copy numbers of unknown replicates from three separate runs was used to estimate the interassay variation.

### Statistical analysis.

Boxplots were built with Rstudio (v.1.2.5001), and Excel 2016 (v.16.0.4964.1000) was used to create scatterplot graphs. Pearson correlation coefficient between estimated and quantified copies in virus mixes was calculated with SPSS (v.25). Venn diagrams were made with InteractiVenn (66).

### Ethics statement.

The Ethics Committee of the Helsinki and Uusimaa Hospital District approved the collection of tonsils. Informed consent was obtained from all the donors or their parents prior to the surgery.
