# Supplementary material for: HERQ-9 Is a New Multiplex PCR for Differentiation and Quantification of All Nine Human Herpesviruses
Source: mSphere. 2020 Jun 24;5(3):e00265-20. doi: 10.1128/mSphere.00265-20 (PMC7316487; doi:10.1128/mSphere.00265-20)
Supplement: FIG S1 [file mSphere.00265-20-sf001.pdf]

Self-dimers:

```
1 dimer for: EBV FWD
5-cggaagccctctggacttc->
    |||| ||   || ||||
  <-cttcaggtctcccgaaggc-5
```

```
1 dimer for: HHV6A LNA Probe A1
5-acatgttgctagaaagact->
     | || |||| || |
 <-tcagaaagatcgttgtaca-5
```

Cross primer dimers:

```
HSV-1 probeLP1 with VZV probeLP1
HSV-1 probeLP1
5-cgcatacccggaagttcttcagat->
         |   ||| || |||| |
       <-gtgtttcgagcagtcaagcgc-5
```

```
VZV REVLP1 with VZV probeLP1
VZV REVLP1
5-cgcatacccggaagttcttcagat->
         | || | |||| |
       <-gtccctaaagacggtaca-5
```

```
HHV8 fwd 3.1 with H8 Probe 300
HHV8 fwd 3.1
5-cggaggagctagcgtcaatca->
             | |||| |
           <-ctcagtcacagcggcatata-5
```

```
H6B FOTY1 with H6B ROTY 1
H6B FOTY1
5-ggattcaggaaaaaggttctaa->
      |||| ||      || ||
   <-gagtcgttgaggacagttt-5
```

```
HHV6A FWD1-3 with HHV6A REV 10
HHV6A FWD1-3
5-tgtcccttcaactactgaatc->
        | |||||   ||  |
     <-aatgttgagacctccggc-5
```

```
H6B PROBE MVP with HHV7 1. REV
H6B PROBE MVP
5-aggaagcgtttcggtacacttggag->
                |||| | ||
             <-aatgtcatcccaaccacatacg-5
```
