# Supplementary figures and images for: HERQ-9 Is a New Multiplex PCR for Differentiation and Quantification of All Nine Human Herpesviruses
Source: mSphere. 2020 Jun 24;5(3):e00265-20. doi: 10.1128/mSphere.00265-20 (PMC7316487; doi:10.1128/mSphere.00265-20)

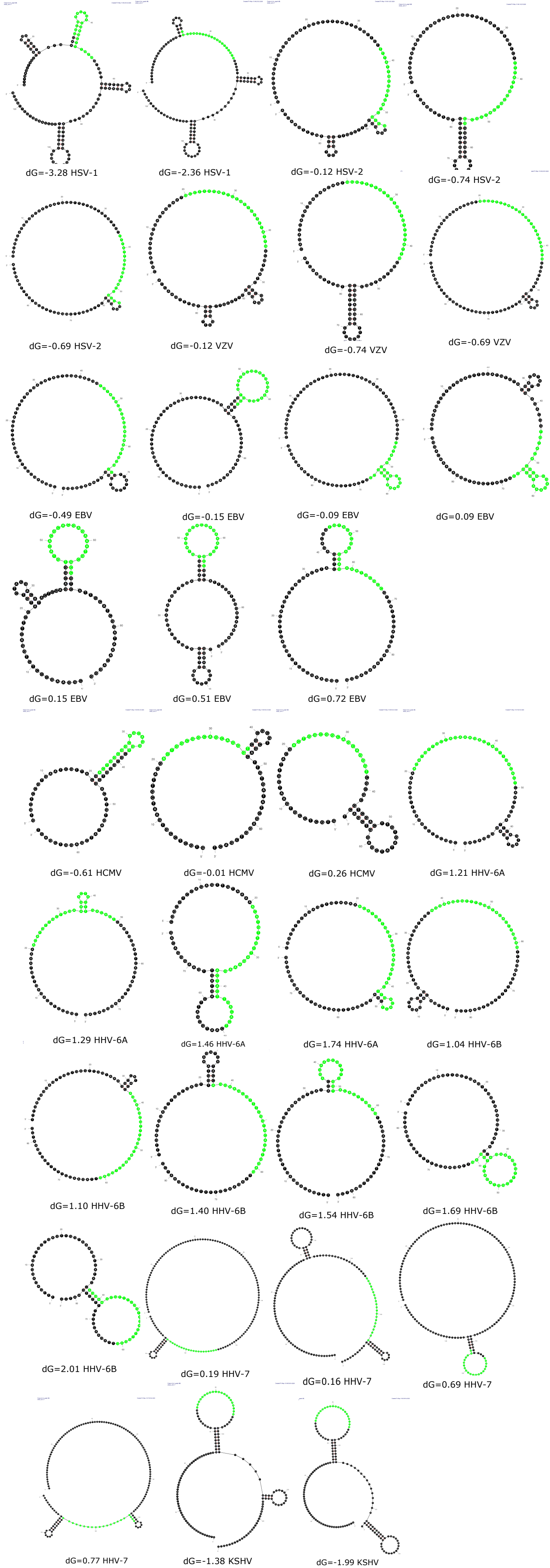

Supplement: FIG S2 [file mSphere.00265-20-sf002.pdf]

## Singleplex

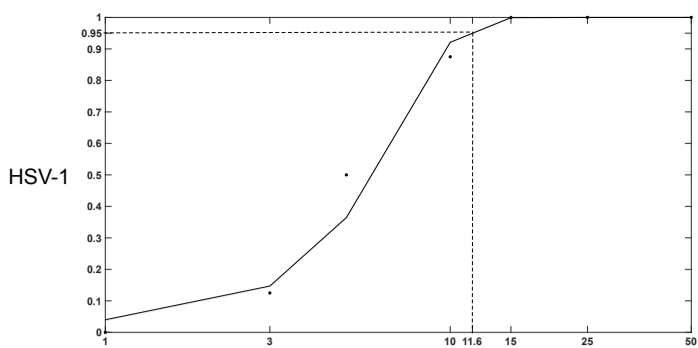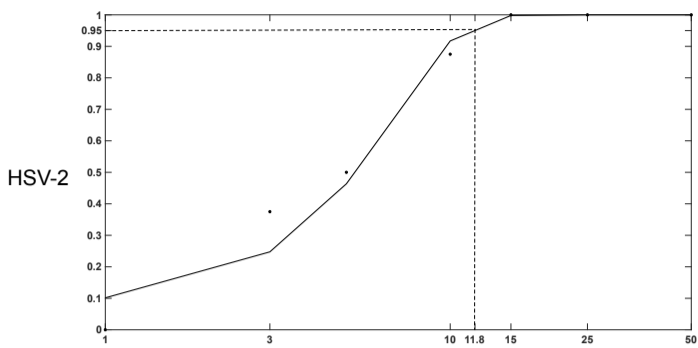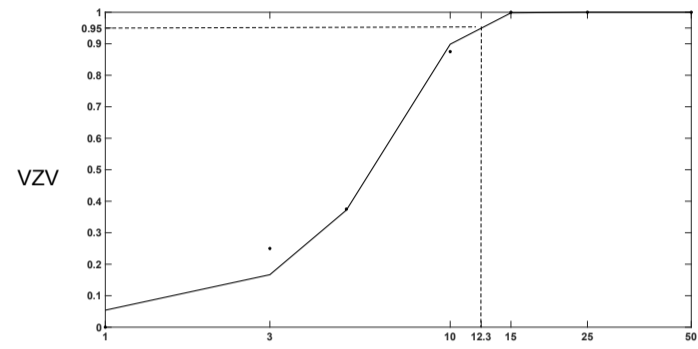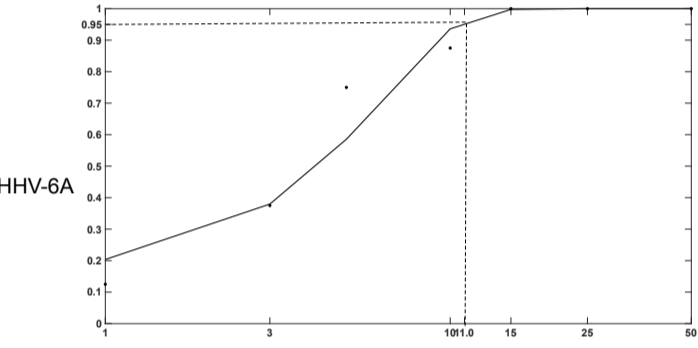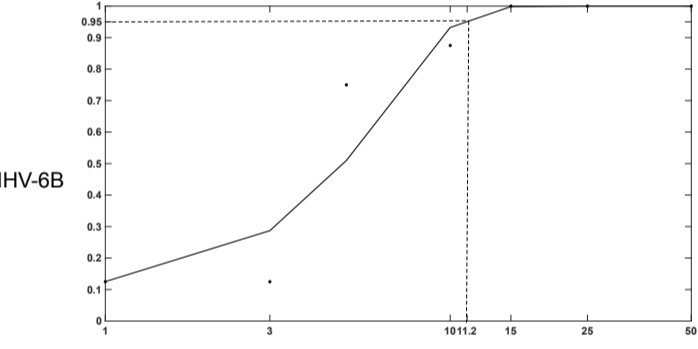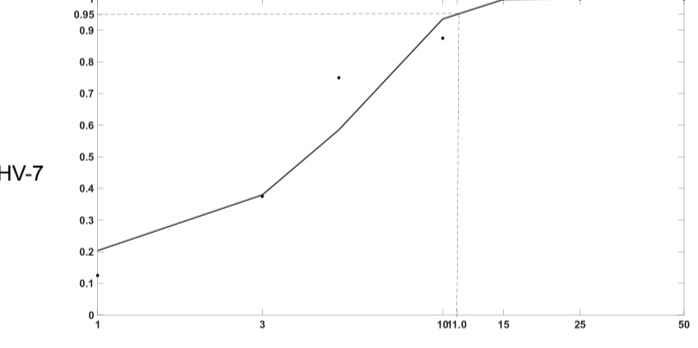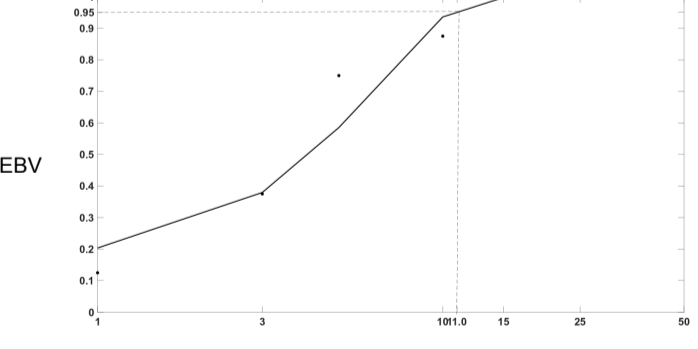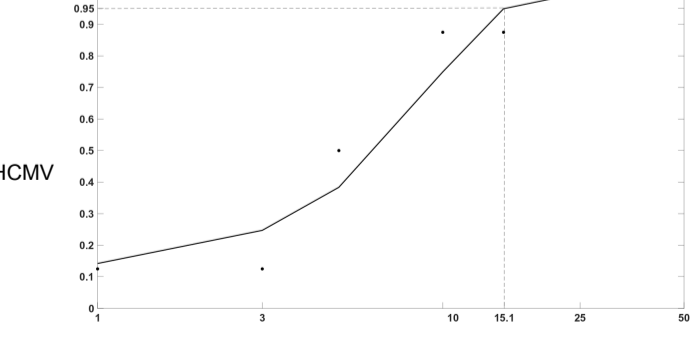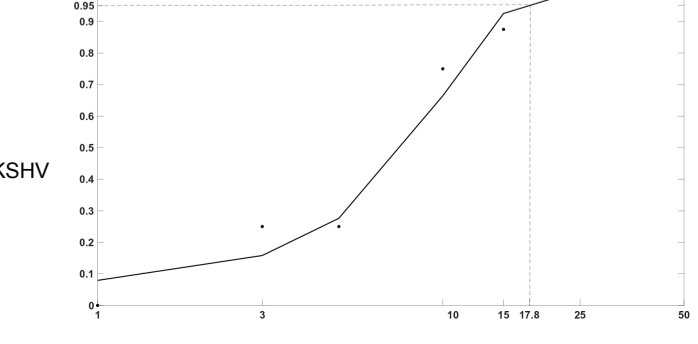

## Multiplex

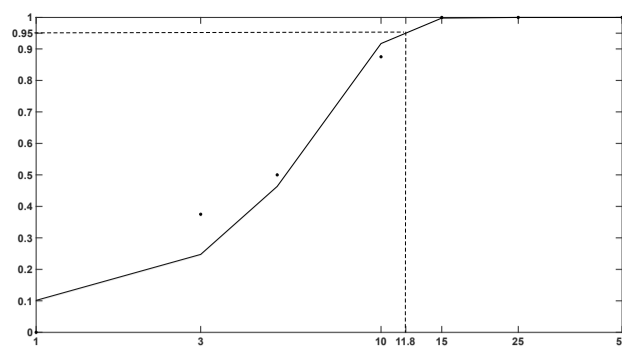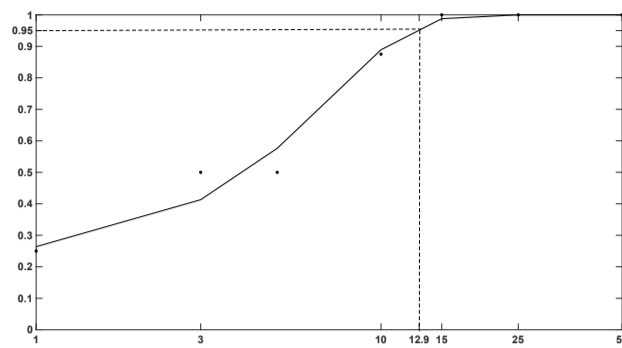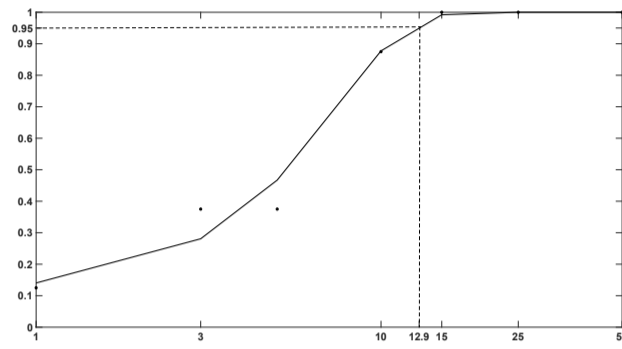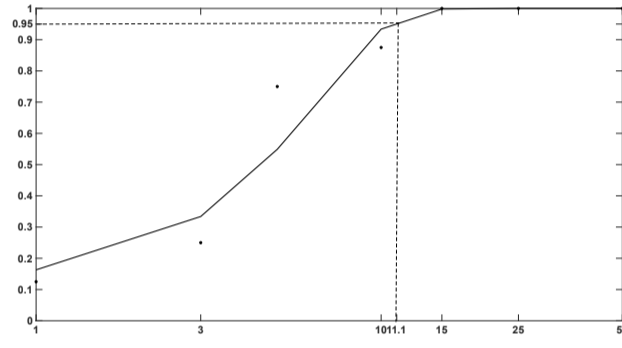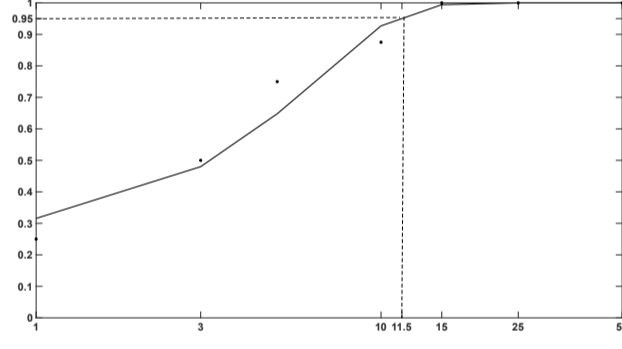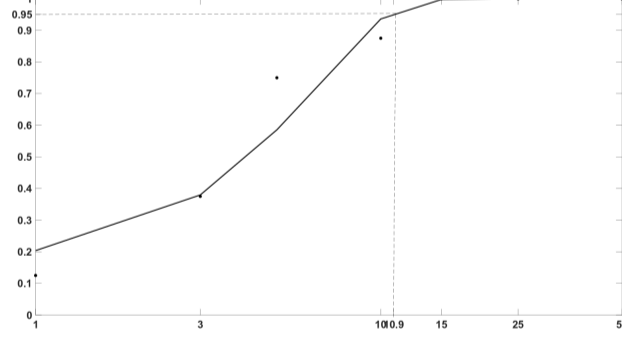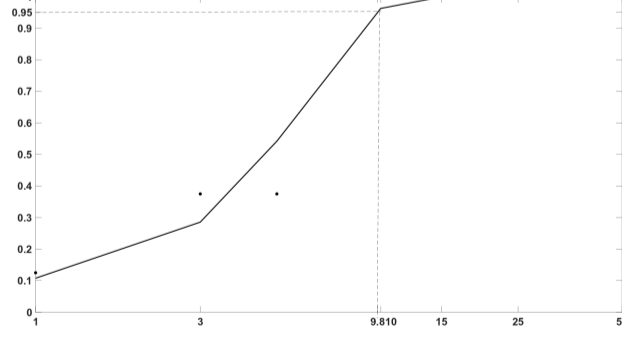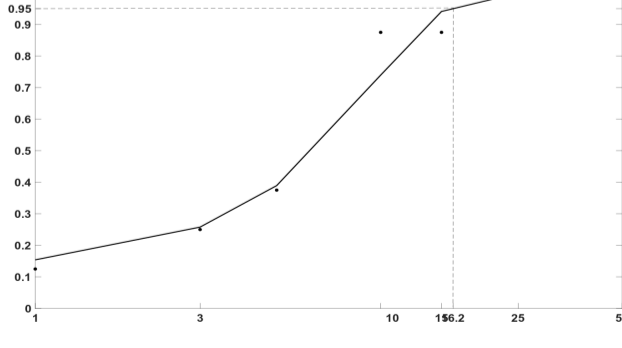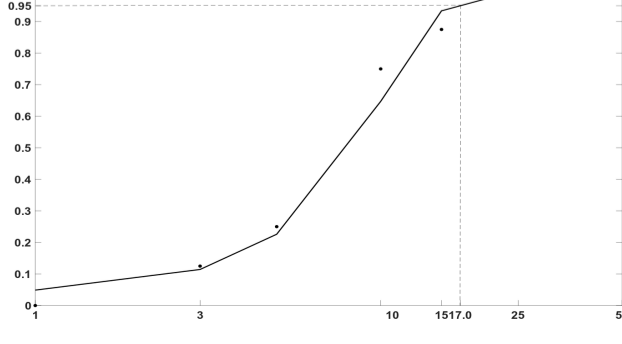

Supplement: FIG S3 [file mSphere.00265-20-sf003.pdf]

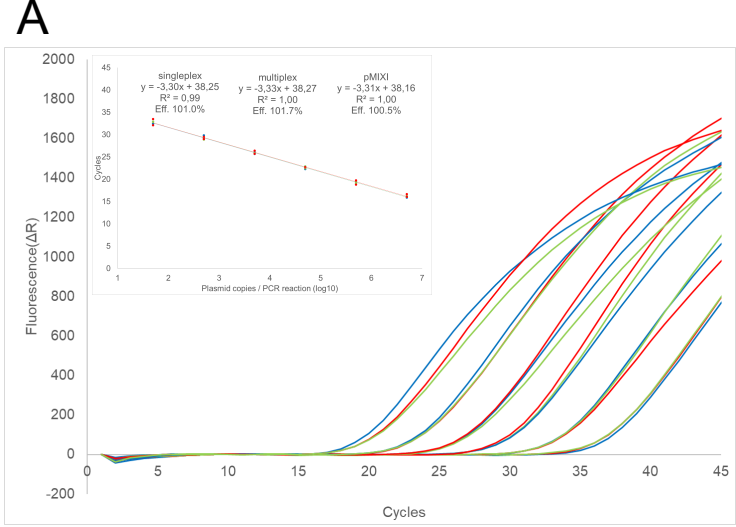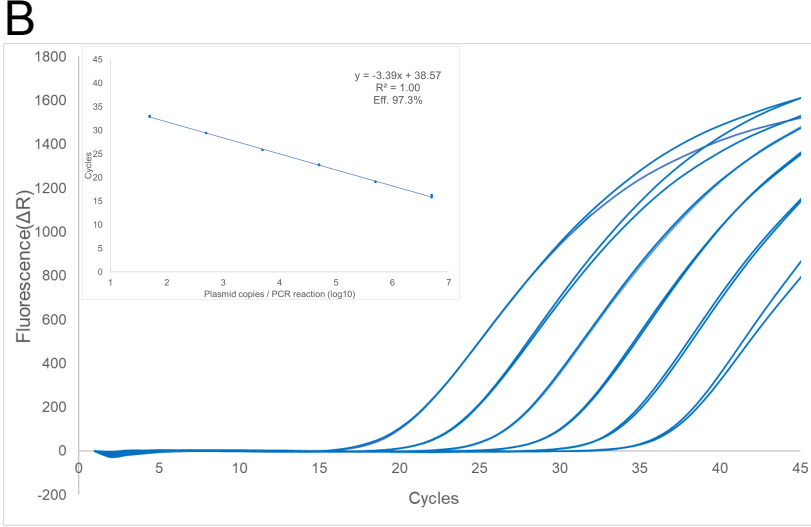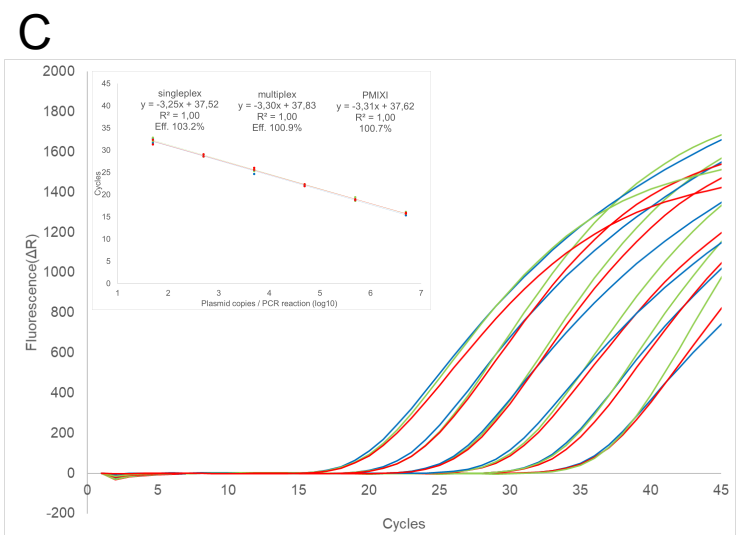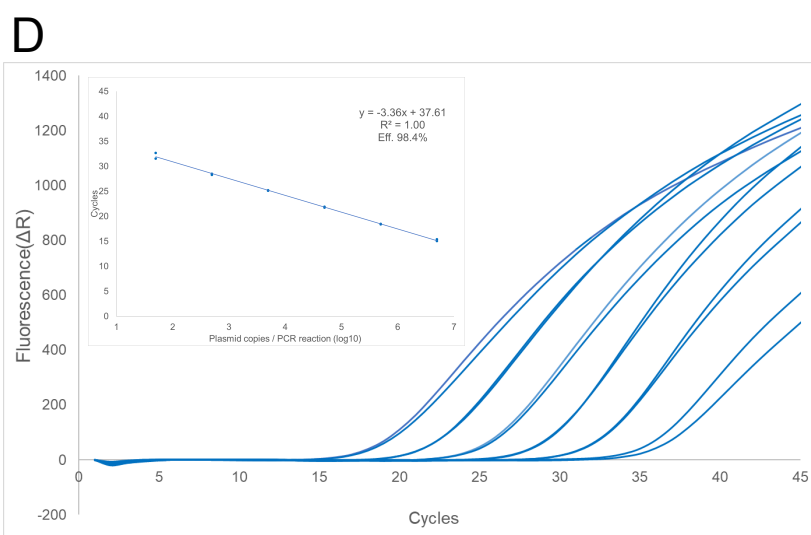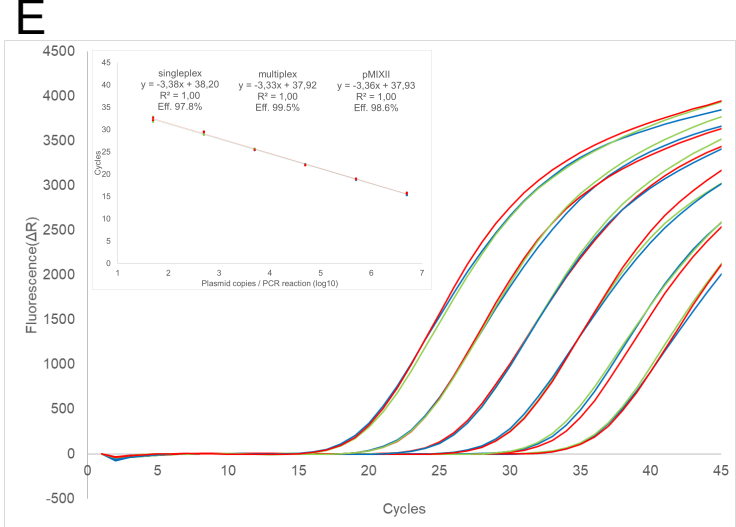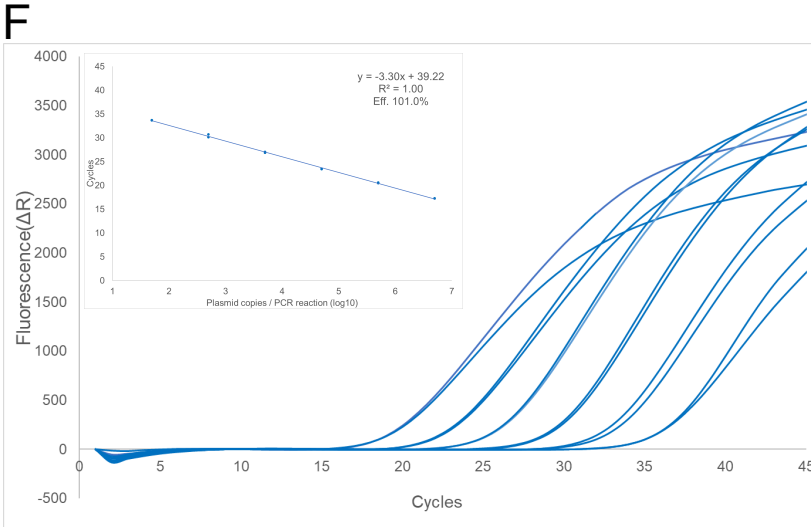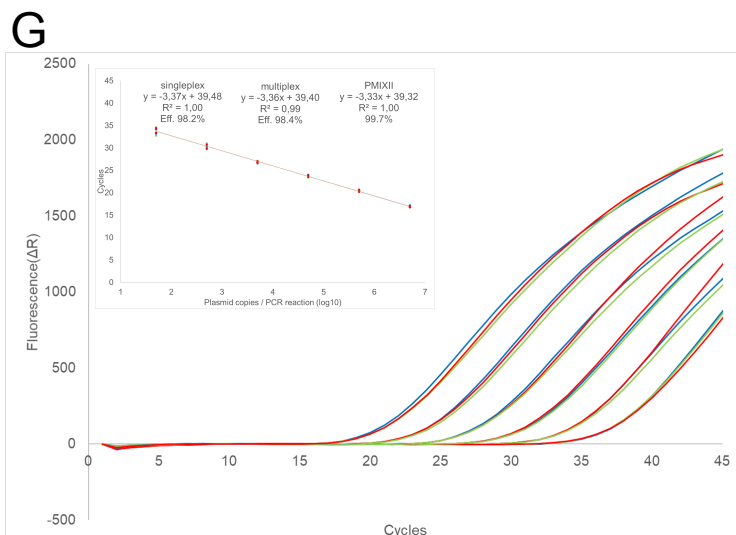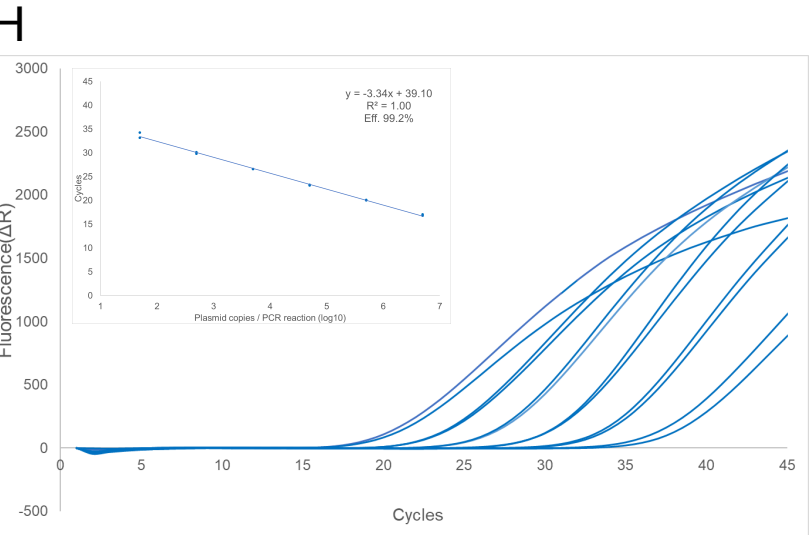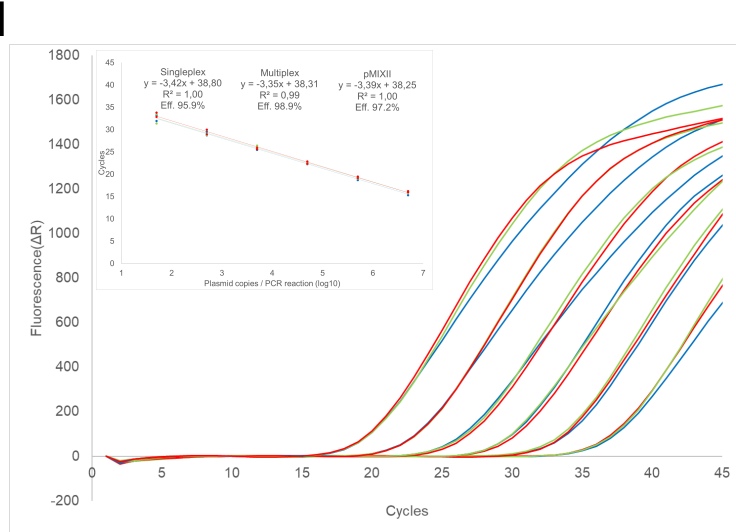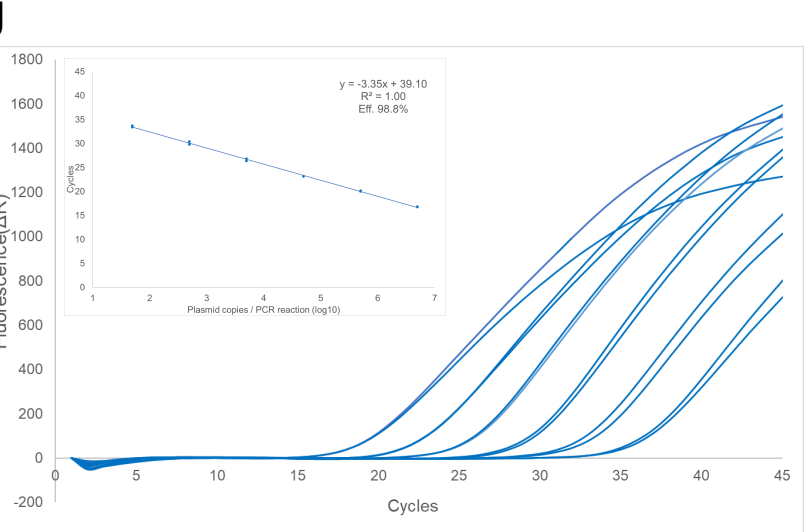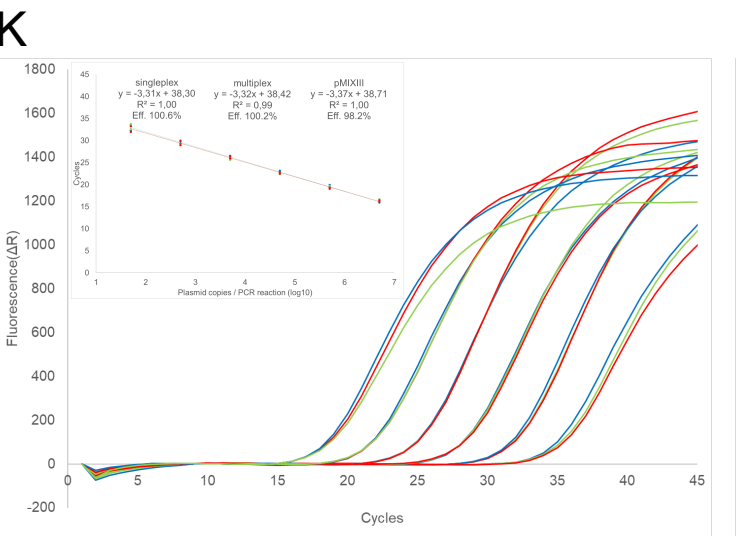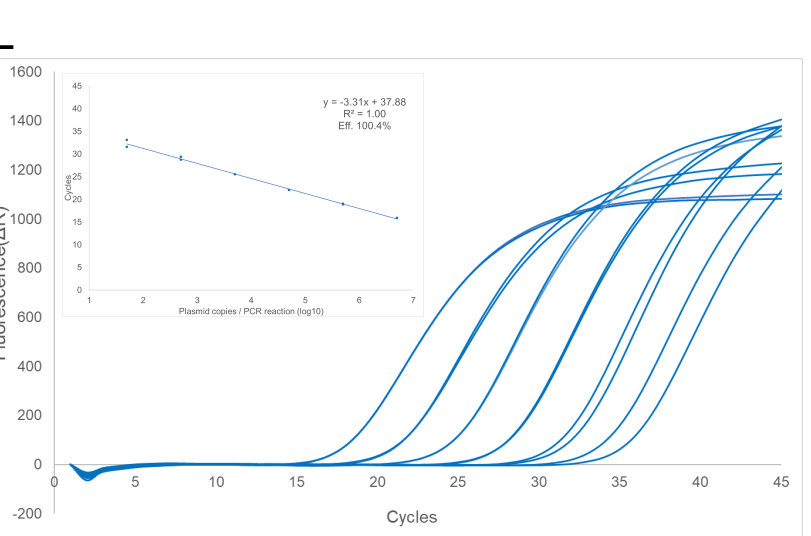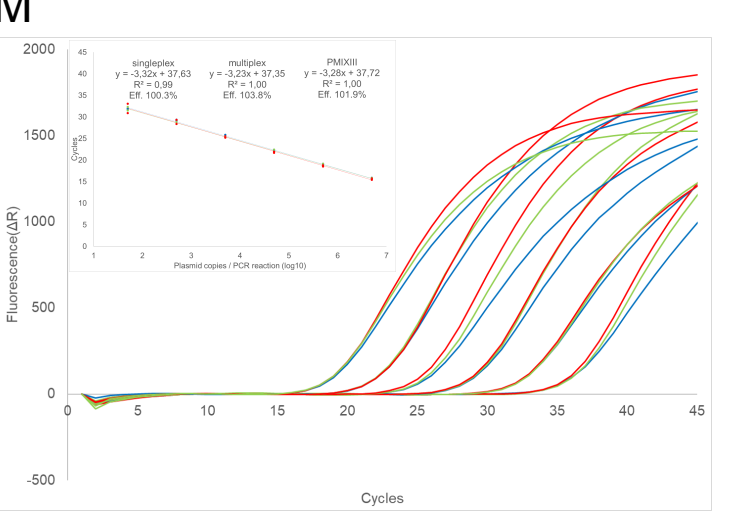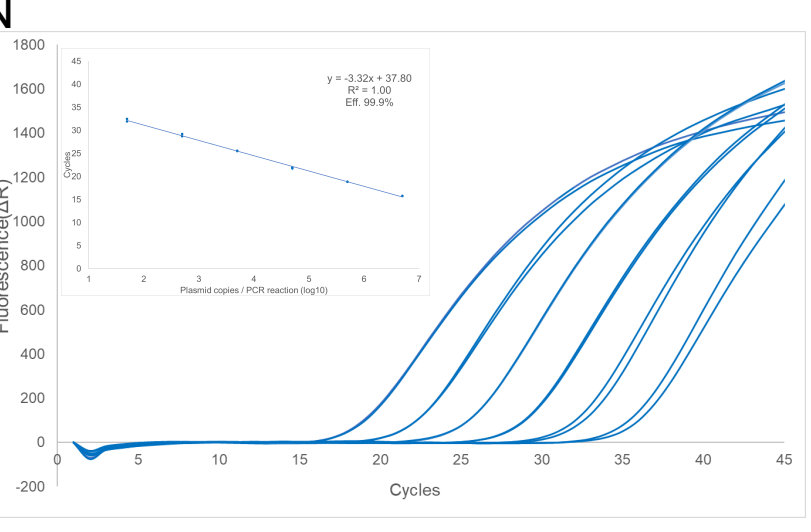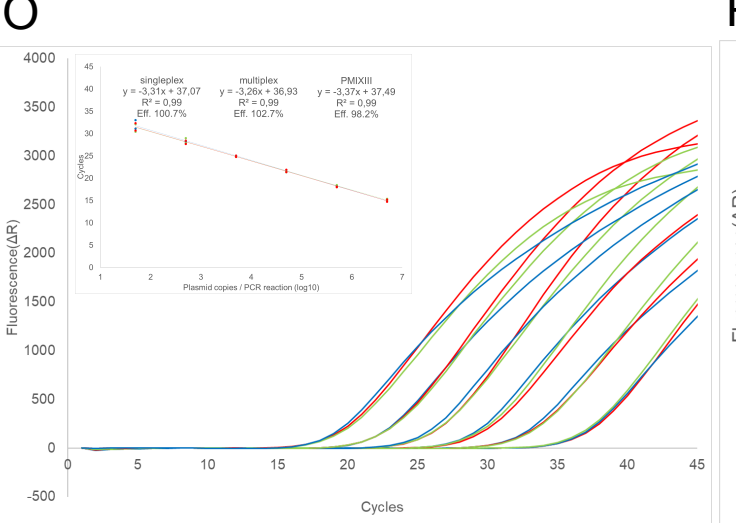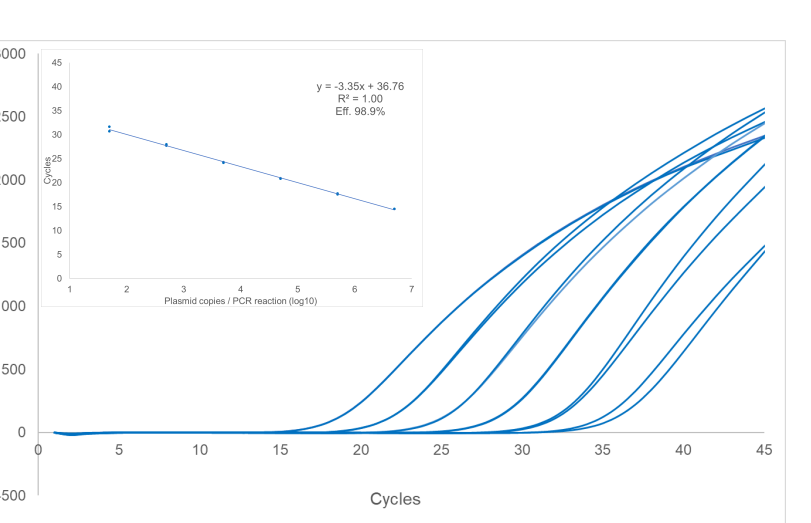

Supplement: FIG S4 [file mSphere.00265-20-sf004.pdf]
