# Supplementary material for: HERQ-9 Is a New Multiplex PCR for Differentiation and Quantification of All Nine Human Herpesviruses
Source: mSphere. 2020 Jun 24;5(3):e00265-20. doi: 10.1128/mSphere.00265-20 (PMC7316487; doi:10.1128/mSphere.00265-20)
Supplement: TEXT S1 [file mSphere.00265-20-s0001.docx]

Sequences of reference strain in the plasmids

**pHSV1**: GCCGTTGAGCTAGCCAGCGAGACGCTGATGAAGC

GCGAACTGACGAGCTTTGTGGTGCTGGTTCCCCAGGGAACCCCCGACGTTCAGTTGCGCCTGACGAGGCCGCAGCTCACCAAGGTCCTTAACGCGACCGGGGCCGATAGTGCCACGCCCACCACGTTCGAGCTCGGGGTTAACGGCAAATTTTCCGTGTTCACCACGAGTACCTGCGTCACCTTTGCTGCCCGCGAGGAGGGCGTGTCGT

**pHSV2**: GTAAGCGCGGGCCAAAGGATGGGGTGGGGTGAGGGTAAAAGCACAAAGGGAGTACC

AGACCGAAAACAAGGACGGATCGGCCCGCTCCGTTTTTCGGTGGGGTGCTGATACGGTGCCAGCCCTGGCCCCGAACCCCCGCGCTTATGGACACACCACACGACAACAATGCCTTTTATTCTGTTCTTTTATTGCCGTCATCGCCGGGAGGCCTTCCGTTCGGGCTTCCGTGTTTGAACTAAACTCCCCCCACCTCGCGGGCAAACGTGCGCGCCAGGTCGCGTATCTCGGCGATGGACCCGGCGGTTGTGACGCGGGTTGGGATCATCCCGGCGGTGA

**pVZV**:GCGCTCTAACGTTCGAGAAAGTCTGCTGGGTGTTCTTTTAAAAGACTGGTTGGCTATGCGCAAGGCTATTAGAGCGCGCATACCCGGAAGTTCTTCAGATGAAGCAGTGTTATTAGACAAACAACAAGCCGCGATAAAAGTAGTTTGTAATTCCGTGTACGGTTTTACTGGAGTTGCGCAGGGATTTCTGCCATGTTTATACGTAGCGGCCACTGTCACTACAATTGGCCGTCAAATGTTATTAAGTACCAGAGATTATATTCA

**pEBV**:CCCTGTTTATCCGATGGAATGACGGCGCATTTCTCGTGCGTGTACACCGTCTCGAGTATGTCGTAGACATGGAAGTCCAGAGGGCTTCCG

**pHCMV**:GCTGACGCGTTTGGTCATCGATCGGCGGATCACCACGTTCGGCTGGTGCTCCGTGAATCGTTACGACTGGCGGCAGCAGGGTCGCGCGTCGACTTGTGATATCGAGGTCGACTGCGATGTC

TCTGACCTGGTGGCCGTGCCCGACGACAGCTCGTGGCCGCGCTATCGATGCCTGTCCTTCGATATCGAGT

**pKSHV**:TATCGGAAGTCACTGGAGTAATGACAACCTCGCAGATTGCTTGTTGGCCATGTAAAAGTATTGATTGTGATTGCTTATTTATCTTACCGAAAGAAAGGATGTTTAAAGCATCGGTTTCGGAAGGATTGGGTTTTTCAATGCCAACGTGATGCCTAACCCAACTATTTACAGTAGGGTTGGTGTATGCGTGCATTGGAAAGACCGAAAATA

**pHHV-6A**: CACTGAAAACAGCAGACAGTCAGGGGACGAACAAACAAACCCTAACTGTGTAGGTACAGCCTCAGTGACAGATCTGGGCGGCCCTGATAACTTGAACTCCATCAGCGGCCTCCAGAGTTGTAAAAACATGTTGCTAGAAAGACTACTTGACACTCAATGCGATTCAGTAGTTGAAGGGACAGAACAAG

**pHHV-6B**: CACTGAAAACAGCAGACAGTCAGGGGACGAACAAACAAACCCTAACTGTGTAGGTACAGCCTCAGTGACAGATCTGGGCGGCCCTGATAACTTGAACTCCATCAGCGGCCTCCAGAGTTGTAAAAACATGTTGCTAGAAAGACTACTTGACACTCAATGCGATTCAGTAGTTGAAGGGACAGAACAAG

**pHHV-7** TATCGGAAGTCACTGGAGTAATGACAACCTCGCAGATTGCTTGTTGGCCATGTAAAAGTATTGATTGTGATTGCTTATTTATCTTACCGAAAGAAAGGATGTTTAAAGCATCGGTTTCGGAAGGATTGGGTTTTTCAATGCCAACGTGATGCCTAACCCAACTATTTACAGTAGGGTTGGTGTATGCGTGCATTGGAAAGACCGAAAATA
