# Supplementary material for: HERQ-9 Is a New Multiplex PCR for Differentiation and Quantification of All Nine Human Herpesviruses
Source: mSphere. 2020 Jun 24;5(3):e00265-20. doi: 10.1128/mSphere.00265-20 (PMC7316487; doi:10.1128/mSphere.00265-20)
Supplement: TEXT S2 [file mSphere.00265-20-s0002.docx]

Preparation of HSV-1 and 2 nucleocapsids

The HSV DNA was prepared from isolated viral nucleocapsids in order to minimize contamination by host cell DNA. Clinical HSV-1 and HSV-2 viral isolates were obtained from anonymous samples from herpes lesions. The viruses were typed as HSV-1 or HSV-2 by an immunoperoxidase-rapid culture assay (Ziegler et al., 1988). The typing was confirmed by a type-specific HSV PCR test (Hukkanen et al., 2000). For viral nucleocapsid DNA preparations, low-passage stocks were used, grown in Vero cells (African green monkey kidney; ATCC). The viral genomic DNA was prepared from isolated viral nucleocapsids as described previously (Bowen et al., 2019; Szpara et al., 2011). Shortly, viral stock collected from the Vero cells was used to infect HaCaT cells (Department of Dentistry, University of Turku (Boukamp et al., 1988) in a roller bottle at a multiplicity of infection (m.o.i.) of 0.1-1 pfu/cell, and the infection was allowed to proceed at +35 °C for 1-3 days. The cells were collected, washed with PBS and suspended to a buffer consisting of 0.125 M KCl, 30 mM Tris pH 7.4, 5 mM MgCl2, 0.5 mM EDTA, 0.5% Nonidet P-40 and 0.6 mM beta-mercaptoethanol. After two consequent extractions with Freon (1,1,2-trichloro-1,2,2-trifluoroethane; Sigma-Aldrich) the extracts were added on gradients of 5% to 45% glycerol and ultracentrifuged at 77 000 x g for 1 h at +4^o^C (Beckman Coulter SW41Ti rotor). Viral nucleocapsids were collected from the bottom of the ultracentrifuge tubes, and the DNA was prepared by proteinase K-SDS treatment, followed by extractions with phenol-chloroform, and was recovered by ethanol precipitation. The DNA content was determined by spectrophotometry (DeNovix DS-11+ spectrophotometer) and used for calculation of the viral genome copy numbers.

Supplemental References:

Boukamp, P., Petrussevska, R.T., Breitkreutz, D., Hornung, J., Markham, A., Fusenig, N.E., 1988. Normal keratinization in a spontaneously immortalized aneuploid human keratinocyte cell line. J Cell Biol 106, 761-771.

Bowen, C.D., Paavilainen, H., Renner, D.W., Palomäki, J., Lehtinen, J., Vuorinen, T., Norberg, P., Hukkanen, V., Szpara, M.L., 2019. Comparison of Herpes Simplex Virus 1 Strains Circulating in Finland Demonstrates the Uncoupling of Whole-Genome Relatedness and Phenotypic Outcomes of Viral Infection. J Virol 93, e01824-18.

Hukkanen, V., Rehn, T., Kajander, R., Sjöroos, M., Waris, M., 2000b. Time-resolved fluorometry PCR assay for rapid detection of herpes simplex virus in cerebrospinal fluid. J Clin Microbiol 38, 3214-3218.

Szpara, M.L., Tafuri, Y.R., Enquist, L.W., 2011. Preparation of viral DNA from nucleocapsids. J Vis Exp.54, 3151.

Ziegler, T., Waris, M., Rautiainen, M., Arstila, P., 1988. Herpes simplex virus detection by macroscopic reading after overnight incubation and immunoperoxidase staining. J Clin Microbiol 26, 2013-2017.
